# Healthcare providers’ knowledge, attitude, and practice towards cervical cancer screening in Sub-Saharan Africa: systematic review and meta-analysis

**DOI:** 10.3389/fonc.2024.1436095

**Published:** 2024-08-19

**Authors:** Amare Mebrat Delie, Eyob Ketema Bogale, Tadele Fentabel Anagaw, Misganaw Guadie Tiruneh, Eneyew Talie Fenta, Destaw Endeshaw, Habitu Birhan Eshetu, Ousman Adal, Abiyu Abadi Tareke, Natnael Kebede

**Affiliations:** ^1^ Department of Public Health, College of Medicine and Health Sciences, Injibara University, Injibara, Ethiopia; ^2^ Department of Health Promotion and Behavioral Science, School of Public Health, College of Medicine and Health Sciences, Bahir Dar University, Bahir Dar, Ethiopia; ^3^ Department of Health Systems and Policy, Institute of Public Health, College of Medicine and Health Sciences, University of Gondar, Gondar, Ethiopia; ^4^ Department of Adult Health Nursing, School of Health Sciences, College of Medicine and Health Sciences, Bahir Dar University, Bahir Dar, Ethiopia; ^5^ Department of Health Promotion and Health Behaviour, Institute of Public Health, College of Medicine and Health Sciences, University of Gondar, Gondar, Ethiopia; ^6^ Department of Emergency and Critical Care Nursing, College of Medicine and Health Sciences, Bahir Dar University, Bahir Dar, Ethiopia; ^7^ Amref Health Africa, West Gondar Zonal Health Department, SLL Project, Gondar, Ethiopia; ^8^ Department of Health Promotion, School of Public Health, College of Medicine Health Sciences, Wollo University, Dessie, Ethiopia

**Keywords:** cervical cancer screening, healthcare provider, knowledge, attitude, practice, sub-Saharan Africa

## Abstract

**Introduction:**

Cervical cancer is a prevalent cancer among women in low and middle-income countries, but it can be largely prevented through screening programs and HPV vaccination. This study aimed to determine the level of knowledge, attitudes, and practices regarding cervical cancer screening among healthcare providers in Sub-Saharan African countries.

**Methods:**

Systematic review and meta-analysis were conducted according to the Preferred Reporting Items for Systematic Reviews and Meta-Analysis guidelines. Relevant databases including PubMed, Cochrane Library, AJOL, Google Scholar, and ScienceDirect databases were used to retrieve and search articles. The study included published and unpublished research written in English between January 2013 and May 16, 2024 for studies reporting knowledge, attitude, and practice towards cervical cancer screening among healthcare providers in Sub-Saharan Africa. This review has been registered on PROSPERO. The heterogeneity of the data was evaluated using the I^2^ statistic. A meta-analysis was conducted using STATA 17 software, with a 95% confidence interval. The researchers also conducted publication bias and sensitivity analysis.

**Results:**

The review included 30 studies involving 7542 healthcare providers. The pooled magnitude of good knowledge status towards cervical cancer was 67.93% (95% CI: 53.36–82.50) whereas the pooled magnitude of positive attitude towards cervical cancer was 55.26% (95% CI: 34.28– 76.23). The results also showed that about 49.68% (95% CI: 33.18–66.17) of healthcare providers had good knowledge status about cervical cancer screening, 66.63%(95% CI: 50.36– 82.89) had a positive attitude towards it, and only 17.23% (95% CI; 6.08-28.37) had ever screened for cervical cancer.

**Conclusion:**

The overall magnitude of knowledge and attitude of healthcare providers in Sub-Saharan Africa towards cervical cancer and its screening was suboptimal. Furthermore, a low percentage of female healthcare providers in the region had undergone screening for cervical cancer. As a result, policymakers and program administrators should focus on improving the knowledge, attitude, and practices of healthcare providers to meet the global health goal of cervical cancer screening and effectively eliminating cervical cancer. Healthcare providers must serve as role models for other women who should also undergo screening.

**Systematic review registration:**

https://www.crd.york.ac.uk/PROSPERO/, identifier CRD42023495241.

## Introduction

Cervical cancer is the fourth most prevalent cancer among women on a global scale, comprising approximately 604,000 new cases and leading to 342,000 deaths worldwide (1). The majority of deaths from cervical cancer occur in underdeveloped or developing countries, accounting for about 85% of the total. In low-income and middle-income countries, the death rate from cervical cancer is 18 times higher compared to wealthier countries (2). The number of new cases and deaths from cervical cancer in sub-Saharan Africa is expected to increase over the next 20 years starting from 2013 (3). In comparison, Northern Africa has made significant progress in reducing the occurrence and death rate of cervical cancer, making it the region with the lowest rates in Africa (4).

The risk factors for cervical cancer consist of human papillomavirus infection (HPV), having multiple sexual partners, starting sexual activity at a young age, giving birth to multiple children, having a low socioeconomic status, and smoking tobacco (5–7). Around 70% of cervical cancer cases can be attributed to HPV types 16 and 18 (8). HPV disrupts the normal activity of cells, causing noticeable alterations in the epithelial cells located in the transformation zone of the cervix (9). It is the most common sexually transmitted infection globally. It is most frequently found in teenage and young adult women, which aligns with the timing of their first sexual experiences (10).

This cancerous disease takes a long time to develop into malignant tumors, with the presence of precancerous lesions indicating the ongoing infection (11). It takes between 10 to 15 years for cervical cancer to develop (12). Cervical cancer is a fatal illness when it becomes invasive, but it can be prevented through effective screening programs by identifying and treating premalignant lesions (13). Although vaccines can provide significant protection against HPV for women who have never been exposed to the virus, such as young girls and teenagers, those who have previously been vaccinated will still need to undergo cervical cancer screening later in life to protect against other strains of HPV not included in the vaccines (14). There are several methods for cervical screening, including Pap smear test, HPV DNA test, visual inspection with acetic acid (VIA), or visual inspection with Lugol’s iodine (VILI) (15–19). Properly identifying precancerous lesions and HPV infection through cervical cancer screening methods could significantly decrease cervical cancer deaths (20).

The World Health Assembly has adopted the Global Strategy for eliminating cervical cancer, aiming to achieve it by 2030. This strategy includes three global targets known as 90-70-90, which aim to have 90% of girls vaccinated against HPV by the age of 15, screen 70% of women aged 35-45, and provide treatment to 90% of women diagnosed with cervical disease (21). This strategy aims to eradicate cervical cancer as a major public health issue worldwide, with a goal of reducing the incidence rate to below 4 cases per 100,000 women per year. A global plan to eliminate cervical cancer by 2030 in low- and lower-middle-income countries is projected to reduce the incidence rate by 42% by 2045 and 97% by 2120, preventing over 74 million new cases. Additionally, it is estimated that 300,000 cervical cancer deaths will be prevented by 2030, with over 62 million deaths averted by 2120 (21). The World Health Organization recommends that women in the general population begin cervical cancer screening at age 30. However, women with HIV should be screened more often (every 3 to 5 years) due to their significantly higher risk of developing cervical cancer. For women who receive negative results on VIA or cytology tests, it is recommended to undergo regular screening every three to five years. In women who test negative on an HPV test, rescreening should be done after a minimum interval of five years (22). Healthcare providers are vital in educating people about the risk factors and prevention of cervical cancer, raising awareness to implement effective screening programs and decrease the number of cases. They are influential in encouraging individuals to seek care and have the expertise to educate them on the disease, its causes, risk factors, and screening options, ultimately impacting their screening behavior (23).

Globally, a substantial 40.0% of women were not aware that HPV is responsible for over 95 percent of cervical cancer cases. Among those uninformed about the HPV-cervical cancer link, a notable 39.1% had refrained from undergoing cervical cancer screening, surpassing the global average of 31.2% (1). Furthermore, 31.2% of individuals worldwide have never undergone cervical cancer screening. A large percentage (39.1%) of women who are unaware of the link between HPV and cervical cancer have never been screened for the disease, which is higher than the worldwide average (1). Specifically, Saudi Arabia (55.8%) and Serbia (36.5%) have the highest percentages of women who have never undergone cervical cancer screening. This discrepancy highlights an awareness gap that influences cervical cancer screening rates (1).

However, not all women undergo cervical cancer screening, with the highest risk being among those who have never been screened for cervical cancer till their diagnosis. Additionally, women without health insurance and recent immigrants are less likely to receive cervical cancer screening (24, 25). The rate at which individuals participate in cervical cancer screening can differ based on their understanding of the disease and screening options, as well as other factors like personal opinions, beliefs, attitudes, cultural influences, and the attitude of their partner (26). Previous studies in sub-Saharan Africa have shown that women face numerous obstacles in accessing cervical cancer screening services, including delayed diagnosis, weak health systems, limited funding, lack of information, high costs, societal and cultural beliefs, low awareness, and lack of clear government policies (27–29).

Around 90% of health workers in Turkey had positive attitudes toward cancer screening tests whereas practice-level screening methods were low, with only 4.2% performing pap smears (30). About 74.6% of women in South India had heard about cervical cancer whereas 76.9% knew about screening methods (31). More than half of the women (62.5%) have a positive attitude towards screening. More than three-fourths of women (349; 86.6%) do not have practice toward cervical cancer screening (31). A recent study conducted in Libya found that healthcare providers in health facilities were not adequately informing women about cervical cancer screening (32).

Although there were several studies conducted regarding knowledge, attitudes, and practice level towards cervical cancer screening among healthcare providers in Sub-Saharan Africa, there is a lack of consistent evidence regarding these healthcare providers’ knowledge, attitudes, and practices. The magnitude of good knowledge, positive attitudes, and ever been practices regarding cervical cancer screening among healthcare providers in Sub-Saharan Africa varies significantly, with rates ranging from as low as 4.89% (33) to as high as 97.4% (34) for knowledge, 30.7% (35) to 97.4% (34) for attitudes, and 8.7% (35) to 72.6% (36) ever screened for cervical cancer. Thus, this review aimed to assess knowledge, attitudes, and practices related to cervical cancer screening among healthcare providers in Sub-Saharan Africa. It is crucial to regularly evaluate and address any gaps in their knowledge as having sufficient knowledge is vital for fostering positive attitudes and effective practices. Healthcare professionals are seen as examples to others and have the responsibility to educate the communities they work with about cervical cancer (37). The results of this study will be utilized by policymakers and program planners to enhance and assess cervical cancer screening services and strategies. The study also will provide baseline information for the future.

## Materials and methods

### Information sources and search strategy

Relevant databases include PubMed, Cochrane Library, AJOL, Google Scholar, and Science Direct. We searched articles from January 2013 and May 16, 2024, that were published in peer-reviewed journals or filed as completed dissertations with observational study design. We used a search strategy by combining the following key terms: knowledge, attitude, practice, cervical cancer, uterine cervical neoplasms, cervical cancer screening, health care provider, health professional, health personnel, health care workers, and Sub-Saharan Africa. We used both free texts, OR, AND, Boolean, and Medical subject heading [MeSH]terms in our search. Also, Gray literature of observational studies and official websites of international and local organizations and universities were searched. The study protocol was registered on PROSPERO, with the registration number CRD42023495241.

### Study inclusion and exclusion criteria

This study included English-language research articles and doctoral dissertations conducted in Sub-Saharan Africa between January 2013 and May 16, 2024. The focus was on cross-sectional studies that examined the knowledge, attitude, and practice of healthcare providers toward cervical cancer screening. Only studies published in peer-reviewed journals or completed dissertations were considered. Our review included studies which assessed the knowledge, attitudes, and practices of various healthcare providers in sub-Saharan Africa towards cervical cancer screening, including physicians, nurses, midwives, anesthetists, pharmacy professionals, optometry professionals, laboratory professionals, dentistry professionals, public health officers, and health extension workers. Studies that did not assess the knowledge, attitude, and practice of health care providers towards cervical cancer screening, as well as those conducted outside of Sub-Saharan Africa or not involving health professionals, were excluded. This study excluded case reports, case series, earlier reviews, and qualitative studies on cervical cancer screening uptake. Moreover, articles that were not fully accessible were excluded after attempting to contact the authors via email at least twice.

### Data extraction and data quality assessment

After searching in relevant databases, the study was imported into Endnote version 20.6 and duplicates were removed. Then, three reviewers (AMD, EKB, and TFA) assessed the title and abstract to determine the eligibility of the article for full-text review. The full texts of the remaining papers were downloaded for further analysis, and full-text reviews were conducted. Finally, after applying inclusion and exclusion criteria, eligible studies were exported to Microsoft Excel version 2019 using a standardized data extraction checklist. Data were extracted using a standardized data extraction spreadsheet format prepared in Microsoft Excel. The data abstraction format includes the author/s name, year of publication, study area, study design, sample size, prevalence of knowledge, prevalence of attitude, and prevalence of cervical cancer practice.

The data quality was assessed using Joanna Briggs Institute’s (JBI’s) critical appraisal checklist for those included studies. Three authors (ETF, DE, and MGT) independently assessed the quality of each article. Whenever necessary, another reviewer (AAT) was involved and any discrepancy was resolved through discussion and consensus. In conclusion, studies that scored 6 or higher out of 9 were classified as being of high quality (38). The studies included in the analysis had quality scores ranging from 6 to 9. Besides, studies that had methodological flaws, incomplete reporting of results; or those for which full text was not available were excluded from the final analysis. Study researchers made two separate attempts to contact article authors whenever additional study information was needed (**Table 1**).

**Table 1:** Methodological quality assessment of included studies using the JBI critical appraisal checklist for prevalence studies.

Author	Was the sample frame appropriate to address the target population?	Were study participants sampled appropriately?	Was the sample size adequate?	Were the study subjects and the setting described in detail?	Was the data analysis conducted with sufficient coverage of the identified sample?	Were valid methods used for the identification of the condition?	Was the condition measured in a standard, reliable way for all participants?	Was appropriate statistical analysis used?	Was the response rate adequate, and if not, was the low response rate managed appropriately?	Total score
Oche, MO	Yes	Yes	Yes	Yes	Yes	Yes	Yes	Yes	Yes	9
Ugwu, E. O.	Yes	Yes	Yes	Yes	Yes	Yes	Yes	Yes	No	8
Anyebe, EE	Yes	Yes	No	Yes	Yes	Yes	No	Yes	Yes	7
Kieti, S	Yes	Yes	No	Yes	Yes	Yes	No	Yes	Yes	6
Gebreegziabher, M	Yes	Yes	Yes	Yes	Yes	Yes	Yes	Yes	Yes	9
Addissie, A	Yes	Yes	Yes	Yes	Yes	Yes	Yes	Yes	Yes	9
Aseres, T	Yes	Yes	Yes	Yes	Yes	Yes	Yes	Yes	Yes	9
Dulla, D	Yes	Yes	Yes	Yes	Yes	Yes	Yes	Yes	Yes	9
Ziwu, R	Yes	Yes	Yes	Yes	Yes	Yes	No	Yes	Yes	8
Niyonzimaj, P	Yes	Yes	Yes	Yes	Yes	Yes	No	Yes	Yes	8
Shayo F	Yes	Yes	Yes	Yes	Yes	Yes	No	Yes	Yes	8
Ndizeye, Z	Yes	Yes	Yes	Yes	Yes	No	Yes	Yes	Yes	8
Ifemelumma, CC	Yes	Yes	No	No	Yes	No	No	Yes	Yes	7
Getahun, F	Yes	Yes	No	Yes	Yes	Yes	No	Yes	Yes	7
Mwale, S	Yes	Yes	Yes	Yes	Yes	Yes	No	Yes	Yes	8
Ogunsuyi, G	Yes	Yes	Yes	Yes	Yes	No	No	Yes	Yes	7
Odenusi, AO	Yes	Yes	Yes	Yes	Yes	Yes	Yes	Yes	Yes	9
Omotunde O.	Yes	Yes	Yes	Yes	Yes	No	No	Yes	Yes	7
Ararsa, T	Yes	Yes	Yes	Yes	Yes	Yes	Yes	Yes	Yes	9
Obol, J	Yes	Yes	Yes	Yes	Yes	Yes	Yes	Yes	Yes	9
Melese B	Yes	Yes	Yes	Yes	Yes	Yes	Yes	Yes	Yes	9
Olarinoye, AO	Yes	Yes	Yes	No	Yes	No	No	Yes	Yes	6
Theophil, T	Yes	Yes	Yes	Yes	Yes	Yes	Yes	Yes	Yes	9
Abebaw, E.	Yes	Yes	Yes	Yes	Yes	Yes	Yes	Yes	Yes	9
Logbo-Akey, K	Yes	Yes	Yes	No	Yes	No	No	Yes	Yes	6
Chitha, W	Yes	Yes	Yes	Yes	Yes	Yes	Yes	Yes	Yes	9
Jegede S	Yes	Yes	Yes	Yes	Yes	No	No	Yes	Yes	7
Berhanu, T	Yes	Yes	Yes	No	Yes	Yes	Yes	Yes	Yes	8
Mathivha, L	Yes	Yes	Yes	Yes	Yes	Yes	Yes	Yes	Yes	9
Nyaaba, J	Yes	Yes	Yes	Yes	Yes	Yes	Yes	Yes	Yes	9

Operational definition

Knowledge: Refers to health care providers’ awareness towards cervical cancer screening in Sub-Saharan Africa.

Attitude: Refers to the way of thinking or feeling of health care providers towards cervical cancer screening.

Practice (P). Refers to every screening status of female health care workers for cervical cancer.

Healthcare Providers: All healthcare workers in Sub-Saharan Africa (physicians, Nurses, Midwives, Anesthetists, Pharmacy professionals, Optometry professionals, Laboratory professionals, Dentistry professionals, Public health officers, and health extension workers).

### Data processing and analysis

Data from Microsoft Excel was transferred to STATA software version 17 for further statistical analysis. The heterogeneity of the study results was evaluated using Cochrane’s Q statistics, and I^2^ statistics (39). The results were presented using a forest plot, tables, and graphs. The test results showed significant heterogeneity, so we used a random-effect meta-analysis model to determine the overall knowledge status, attitude, and practice level. Subgroup analyses were conducted based on geographical region to address the possible source of heterogeneity. We also employed various statistical tests, including funnel plot asymmetry, Egger’s test, and Begg’s test, to examine the presence of publication bias (40). Visual inspection of funnel plots can help identify publication bias, but it is not enough to rely solely on subjective interpretations (41). It is recommended to use additional statistical tests, such as Egger’s test (42) and Begg’s test (43), to more accurately assess the presence and severity of publication bias. Both meta-regression and, nonparametric trim and fill analysis were carried out to detect the source of publication bias and to adjust the pooled magnitude of attitude and practice of health providers respectively. The researchers also conducted sensitivity analyses to determine how each study affected the overall findings on knowledge, attitude, and practice toward cervical cancer screening. They did this by excluding one study at a time.

## Results

### Selection of eligible studies

**Figure 1:**
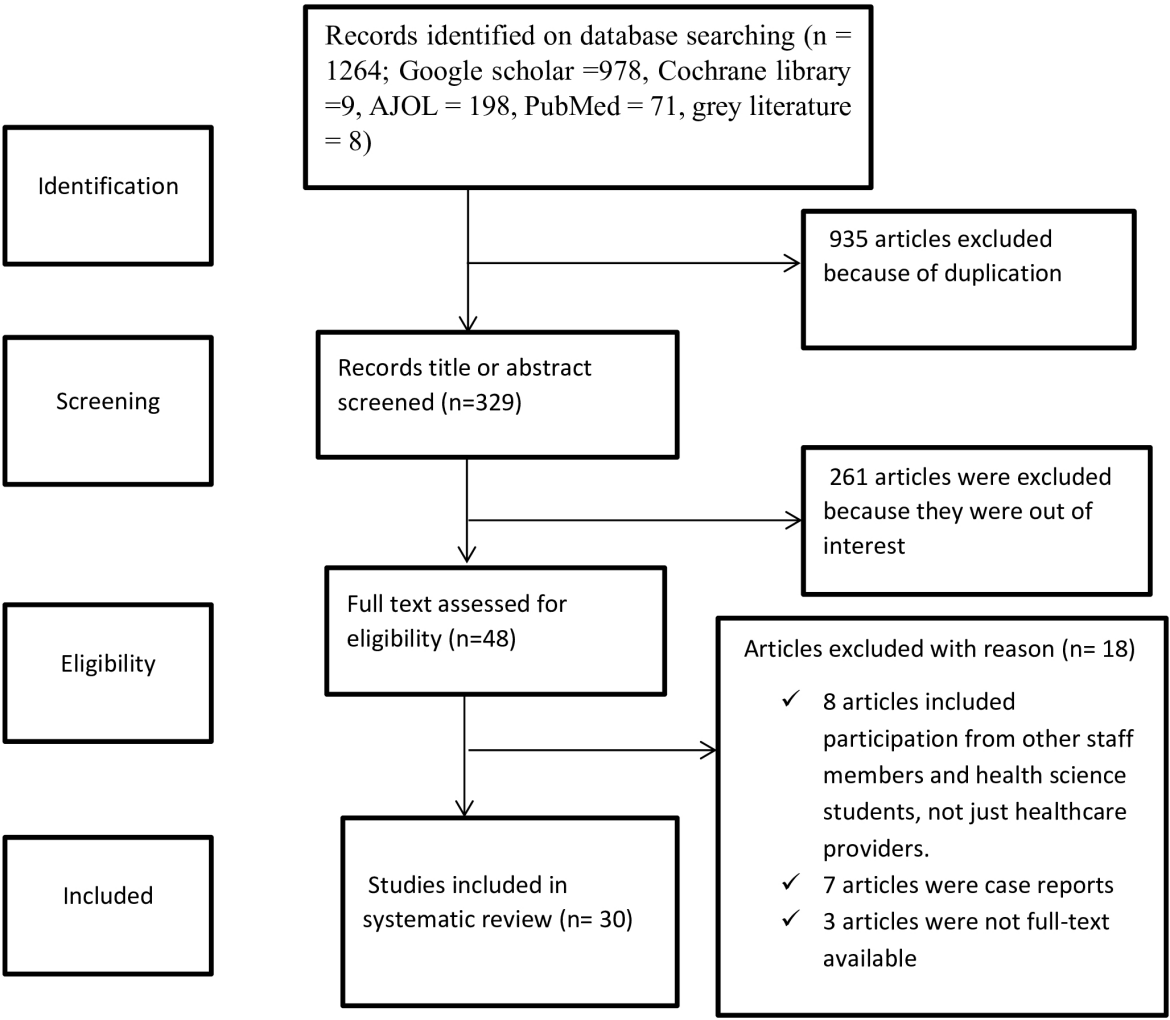
PRISMA flow diagram of knowledge, attitude and practice towards cervical cancer screening among health care providers in Sub-Saharan Africa, 2023.

This systematic review and meta-analysis have been reported by the preferred reporting items for systematic reviews and meta-analyses (PRISMA) statements. Initially, 1264 articles related to knowledge, attitude, and practice towards cervical cancer screening were found. Of these, 935 duplicates and 281 articles by title and abstract were removed. After a thorough review, 18 articles were deemed irrelevant and excluded from the analysis. Ultimately, 30 articles were found to be suitable for the review and were included in the analysis (**Figure 1**).

### Characteristics of included studies

A total of 30 studies with a total sample of 7542 healthcare providers were included in this systematic review and meta-analysis. All the studies in this review were conducted using a cross-sectional study design and were published between 2013 and 2023. Among the 30 studies included, 14 were carried out in Eastern Africa (35, 44–56), 12 studies in Western Africa (23, 34, 57–66), and 4 studies in Southern Africa (67–70). Out of the total number of studies, 7 included both male and female participants (45, 51, 52, 54, 67, 69, 71), while 23 studies only included female healthcare Providers. The pooled magnitude of knowledge status towards cervical cancer screening was determined using 15 studies (34, 35, 46–50, 52, 59–61, 64–66, 69), while the pooled magnitude of attitude towards cervical cancer screening was assessed using 9 studies (34, 35, 47–50, 64, 66, 67). Additionally, 23 studies (23, 34, 35, 45–49, 53–60, 62, 64–66, 68–70) were used to assess the pooled magnitude of practice to ever screened status towards cervical cancer among female healthcare providers in Sub-Saharan Africa. Two studies (51, 71) did not provide the pooled results but instead reported on knowledge status regarding various screening methods such as pap smear, VIA, VILI, colposcopy, and HPV DNA test. Out of the total studies reviewed, 9 were unpublished (46, 49, 52, 53, 56, 58, 64, 67, 70) and the remaining 21 of them were published research (**Table 2**).

**Table 2:** Characteristics of the included studies in meta analysis for knowledge, attitude and practice of healthcare providers towards cervical cancer screening in Sub Saharan Africa, 2023.

Sn	Author	Year	Country	Study Design	Study Population	SS	Knowledge	Attitude	SS For practice	Practice (ever screened)
1.	Oche, MO	2013	Nigeria	CS	FHCW	220	NA	NA	220	10
2.	Ugwu, E. O.	2013	Nigeria	CS	FHCW	177	91	NA	177	14.1
3.	Anyebe, EE	2014	Nigeria	CS	Female nurses	117	97.4	97.4	117	15
4.	Kieti, S	2016	Kenya	CS	FHCW	114	90.35	NA	114	56
5.	Gebreegziabher, M	2016	Ethiopia	CS	Female nurses	225	4.89	63.11	225	10.7
6.	ADDISSIE, A	2016	Ethiopia	CS	FHCW	417	15.9	NA	NA	NA
7.	Aseres, T	2017	Ethiopia	CS	FHCW	322	NA	NA	322	18.3
8.	Dulla, D	2017	Ethiopia	CS	FHCW	367	NA	NA	367	11.4
9.	Ziwu, R	2017	Ghana	CS	FHCW	171	NA	NA	171	16.47
10.	NiyonzimaJ, P	2018	Rwanda	CS	Nurses & midwives	527	NA	NA	464	32.9
11.	Shayo F	2018	Namibia	CS	doctors & Nurses	151	NA	93.4	NA	NA
12.	Ndizeye, Z	2018	Burundi	CS	general practitioners	131	NA	NA	NA	NA
13.	Ifemelumma, CC	2019	Nigeria	CS	female nurses	388	NA	NA	388	20.6
14.	Getahun, F	2019	Ethiopia	CS	HCW	309	NA	NA	NA	NA
15.	MWALE, S	2020	Zambia	CS	female nurses	50	NA	NA	50	25
16.	Ogunsuyi, G	2020	Nigeria	CS	PHCW	192	13	NA	192	NA
17.	Odenusi, AO	2020	Nigeria	CS	FHCW	261	98.4	NA	261	23.2
18.	Omotunde O	2020	Nigeria	CS	Female nurses	407	58.8	48.4	407	23.3
19.	Ararsa, T	2021	Ethiopia	CS	urban HEW	312	48.4	46.8	NA	NA
20.	Obol, J	2021	Uganda	CS	HCW	286	NA	NA	188	75
21.	Melese B	2021	Ethiopia	CS	FHCW	258	21	98.1	258	14.7
22.	Olarinoye, AO	2021	Nigeria	CS	FHCW	348	30.2	NA	348	20.4
23.	Theophil, T	2022	Tanzania	CS	female doctors & nurses	221	NA	NA	221	29.9
24.	Abebaw, E.	2022	Ethiopia	CS	FHCW	404	43.8	30.7	404	8.7
25.	Logbo-Akey, K	2022	Togo	CS	Midwives	50	NA	NA	50	NA
26.	Chitha, W	2023	South Africa	CS	Nurses	119	20.8	NA	106	72.6
27.	Jegede S	2019	Nigeria	CS	FHCW	176	62.4	69.6	176	40
28.	Berhanu, T	2019	Ethiopia	CS	HEW	291	48.5	51.5	291	54
29.	Mathivha, L	2023	South Africa	CS	female nurses	264	NA	NA	264	83
30.	Nyaaba, J	2023	Ghana	CS	FHCW	267	NA	NA	267	35.11

FHCW, Female Health Care Workers; CS, Cross-sectional; HCW, Health Care Workers; HEW, Health Extension Workers; NA, Not Available; PHCW, Primary Health Care Workers; SS, Sample Size.

### Pooled magnitude of knowledge and attitude status of health care providers towards cervical cancer

**Figure 2:**
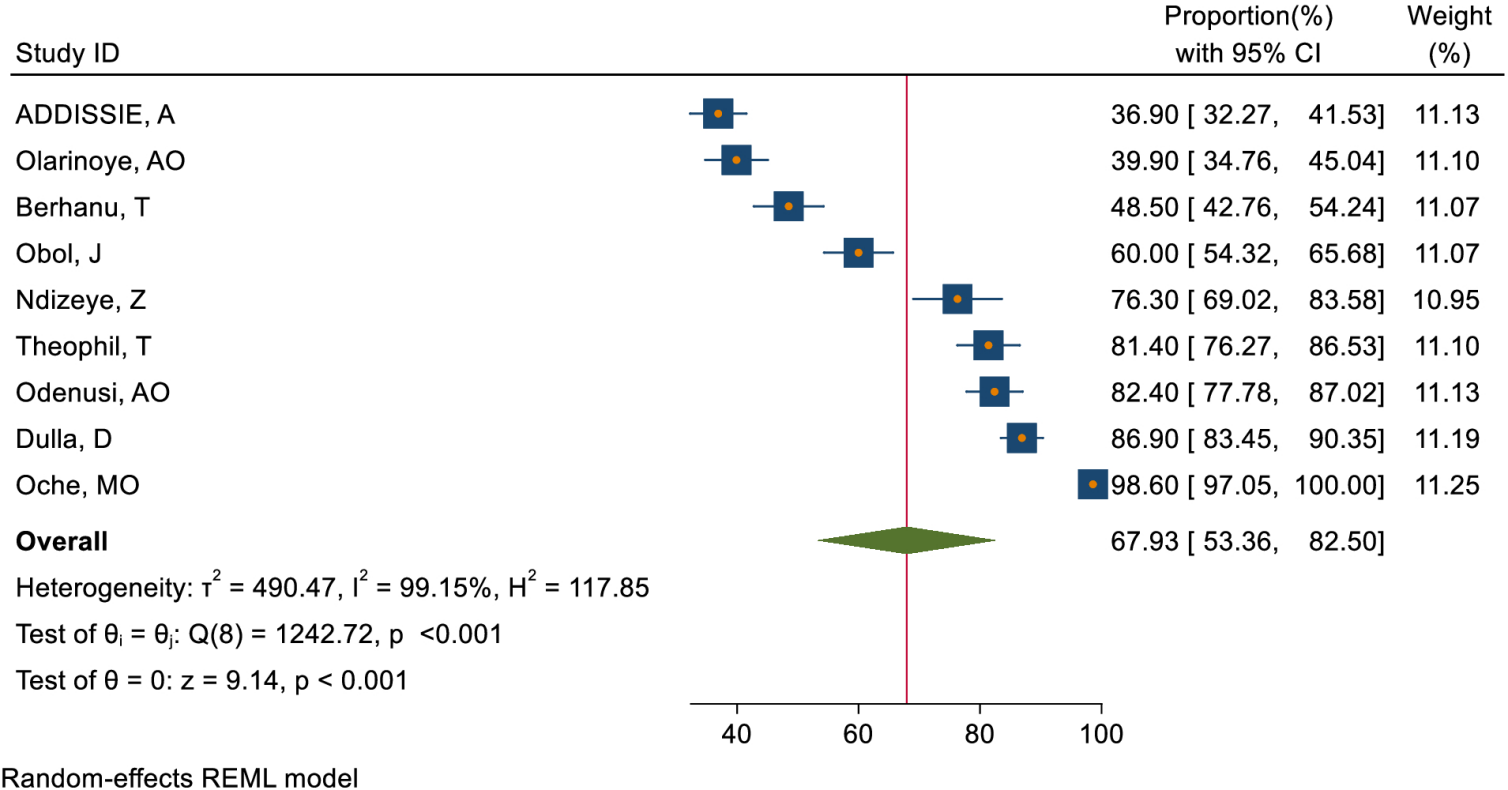
Overall magnitude of goods knowledge status of healthcare providers towards cervical cancer in Sub-Saharan Africa, 2023.

**Figure 3:**
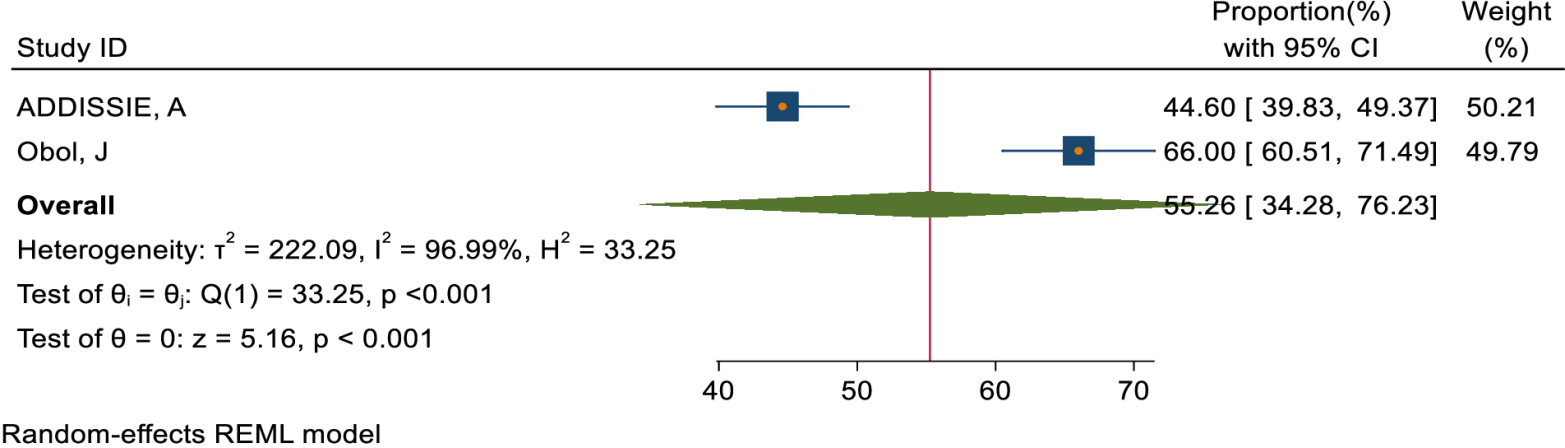
Overall magnitude of positive attitude status of healthcare providers towards cervical cancer in Sub-Saharan African, 2023.

This study assessed the level of knowledge and attitude toward cervical cancer among healthcare providers in Sub-Saharan Africa. Accordingly, the pooled level of good knowledge status about cervical cancer based on 9 included studies (48, 52–55, 57, 59, 60, 71) in Sub-Saharan Africa was 67.93% (95% CI: 53.36–82.50) (**Figure 2**). Moreover, the pooled magnitude of positive attitude of health care providers towards cervical cancer based on 2 included studies (52, 54) in Sub-Saharan Africa was 55.26% (95% CI: 34.28– 76.23) (**Figure 3**).

### Overall knowledge status of health care providers regarding causes, risk factors, signs and symptoms, outcomes, and prevention methods of cervical cancer

**Figure 4:**
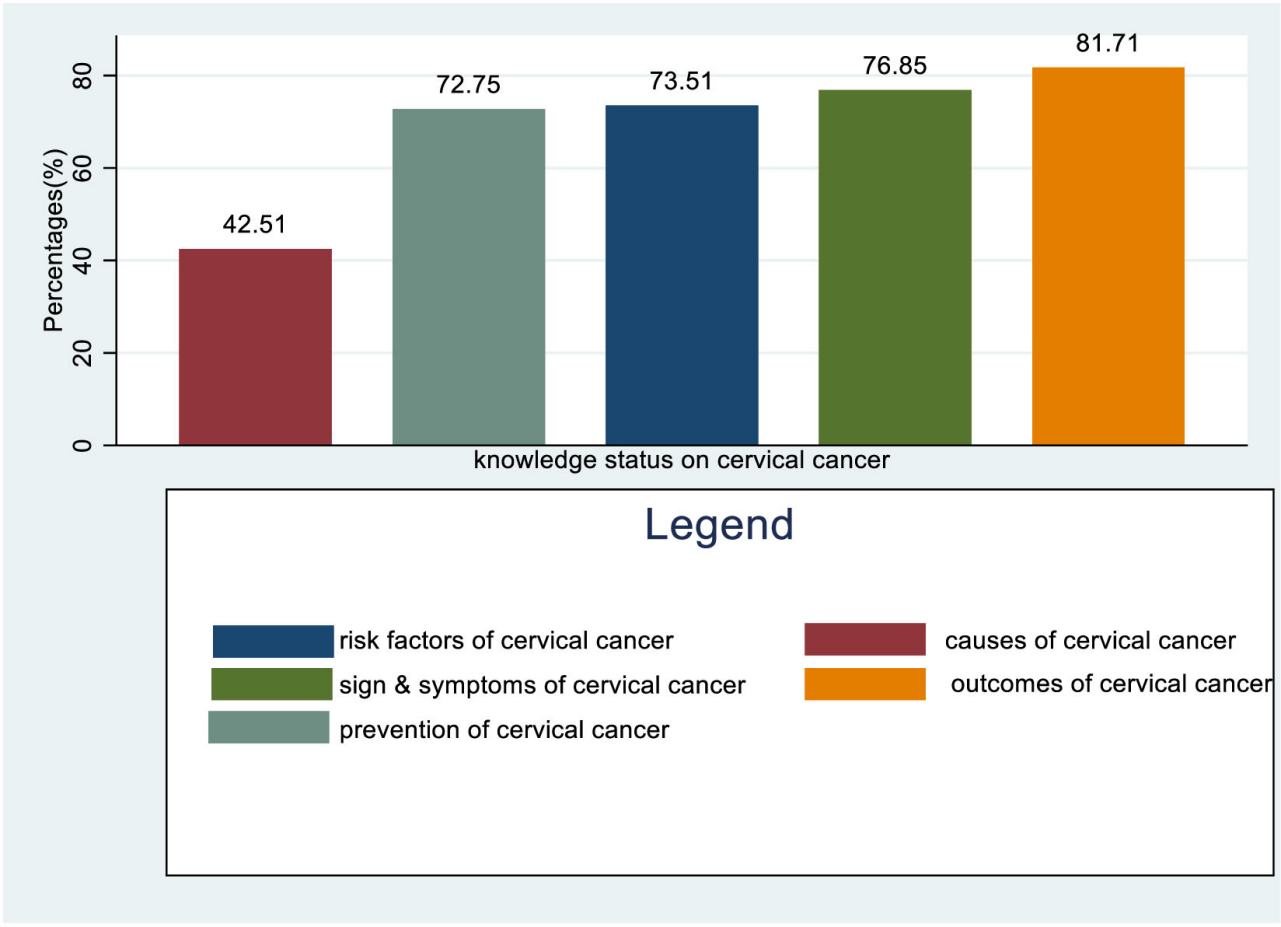
Overall Knowledge status of healthcare providers towards causes, risk factors, symptoms, outcome, and prevention methods of cervical cancer in Sub-Saharan Africa, 2023.

Healthcare providers have a good knowledge status of 42.51% regarding the causes of cervical cancer, 73.51% regarding the risk factors, 76.85% regarding the symptoms, 81.7% regarding the outcomes, and 72.75% regarding the prevention methods (**Figure 4**).

### Knowledge status of health care providers towards risk factors of cervical cancer

**Figure 5:**
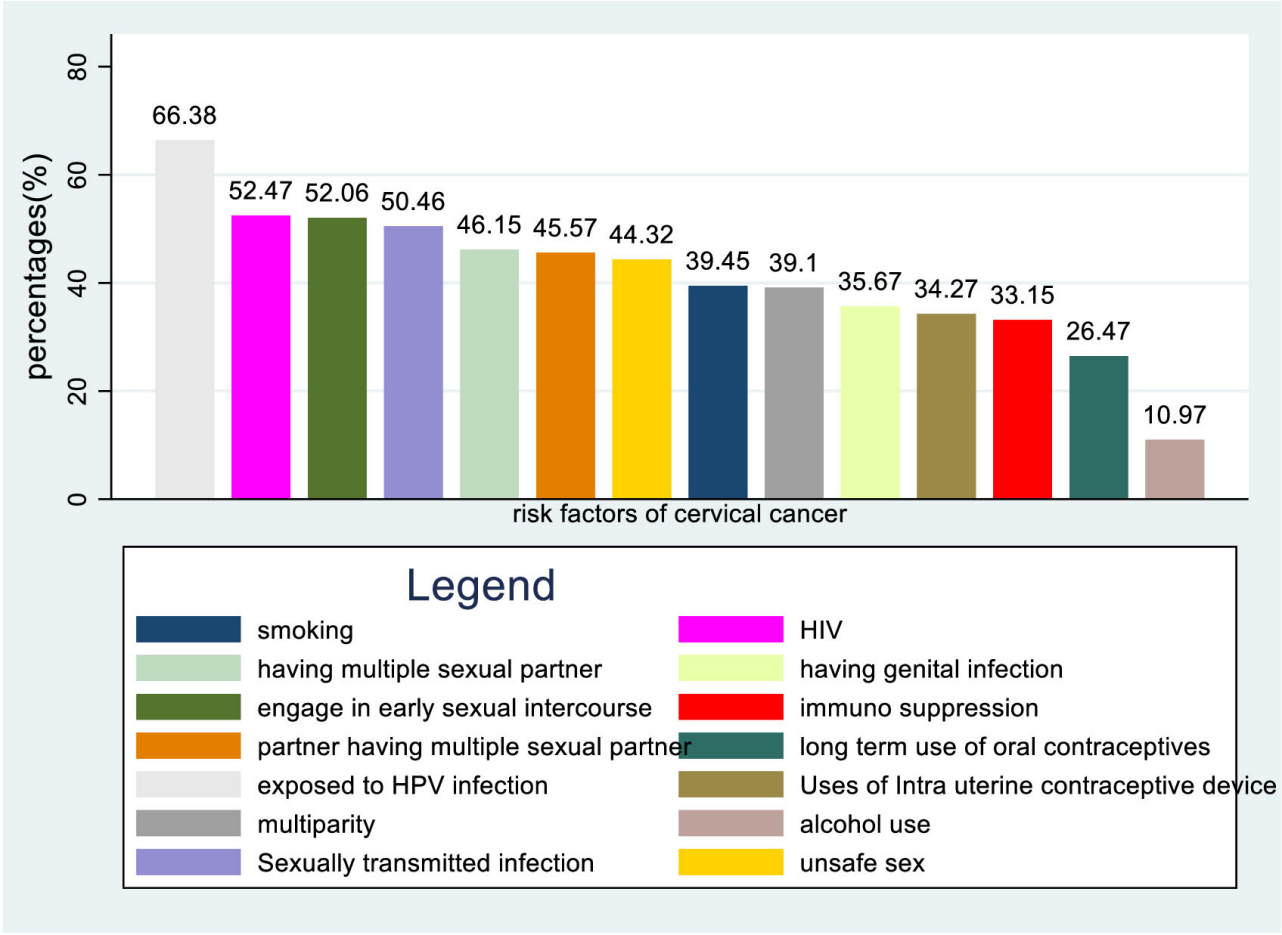
Bar graph for knowledge status of healthcare providers towards risk factors of cervical cancer in Sub-Sahara Africa, 2023.

About 66.38%, 52.47%, 52.06% and 50.46% of healthcare providers know HPV infection, HIV, early sexual intercourse, and STI mention as a risk factor for cervical cancer respectively. In contrast, about 1.98% do not mention any of the risk factors (**Figure 5**).

### Knowledge status of health care providers regarding signs and symptoms of cervical cancer

**Figure 6:**
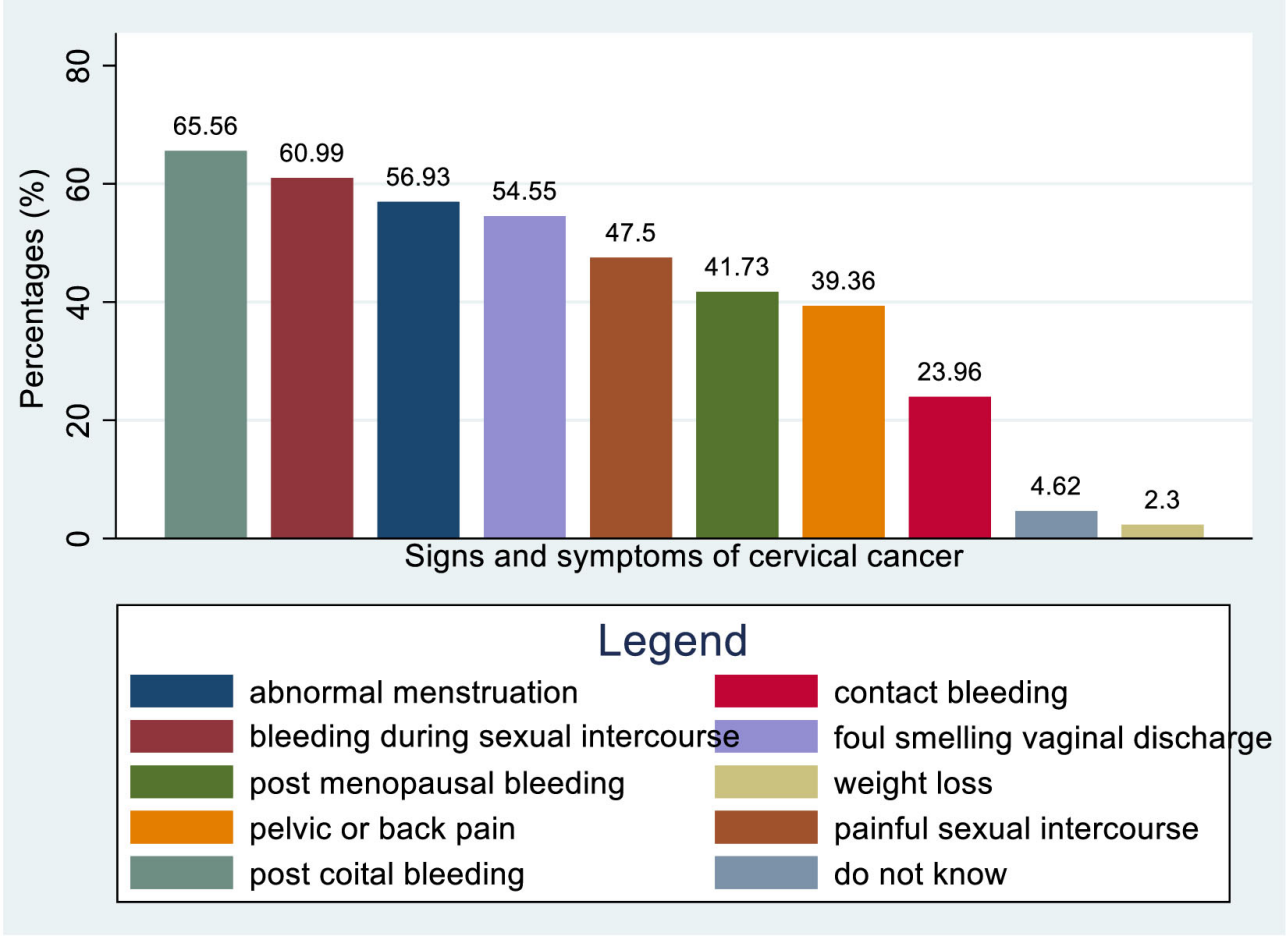
Bar graph for knowledge status of healthcare providers towards symptoms and signs of cervical cancer in Sub-Sahara Africa, 2023.

Around 65.56%, 60.99%, 56.93%, and 54.55% of healthcare providers were aware of postcoital bleeding, abnormal menstruation, bleeding during sexual intercourse, and foul-smelling vaginal discharge as potential signs and symptoms of cervical cancer. Less than half of healthcare providers are knowledgeable about certain symptoms and conditions, such as painful sexual intercourse (47.5%), postmenopausal bleeding (41.73%), pelvic or back pain (39.16%), and contact bleeding (23.96%). A small percentage (4.62%) do not know any of the signs and symptoms, while an even smaller percentage (2.3%) are unaware of weight loss as a potential symptom (**Figure 6**).

### The overall magnitude of knowledge, attitude, and practice status toward cervical cancer screening

**Figure 7:**
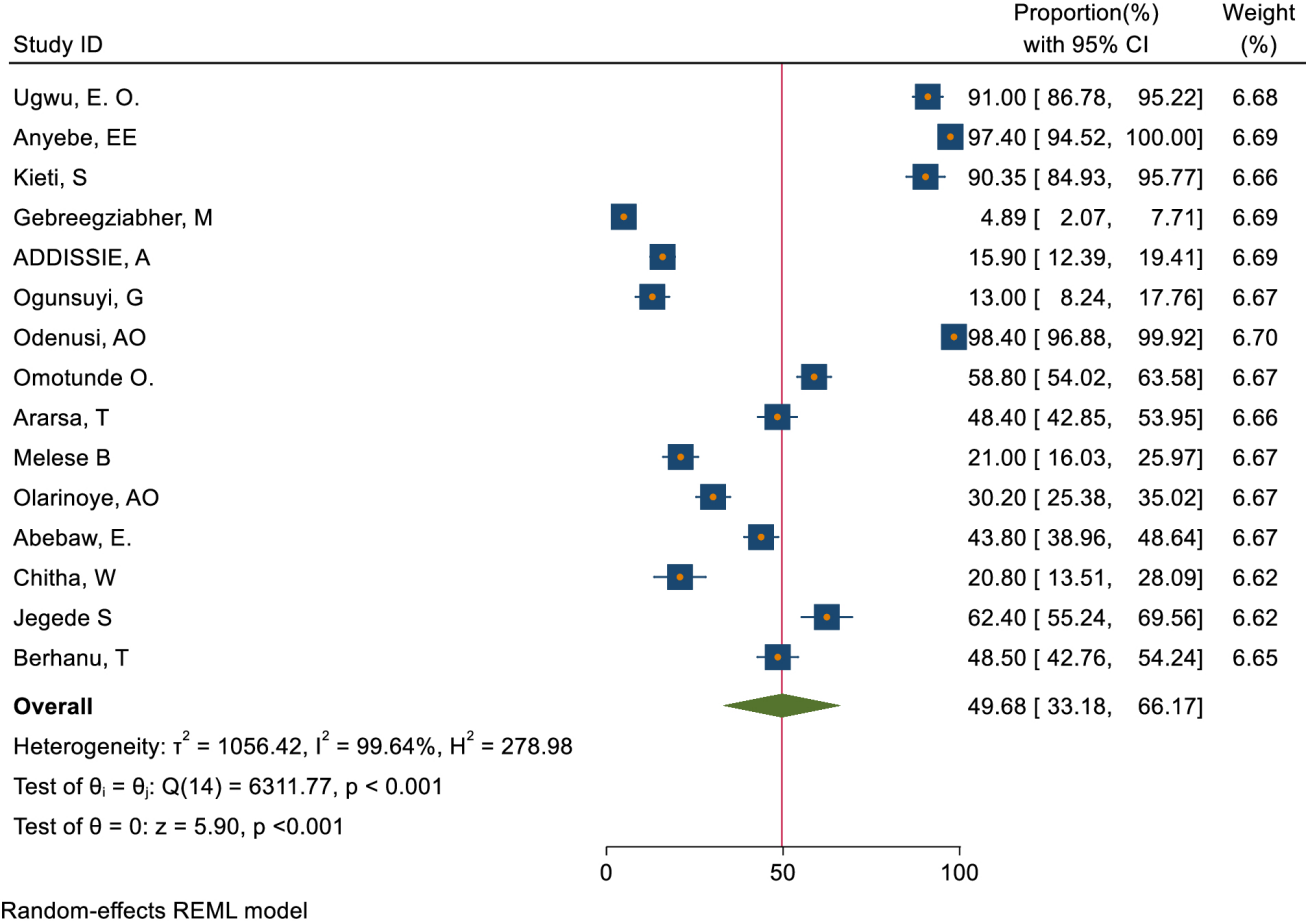
Overall magnitude of good knowledge status of health care providers towards cervical cancer screening in Sub-Saharan Africa, 2023.

**Figure 8:**
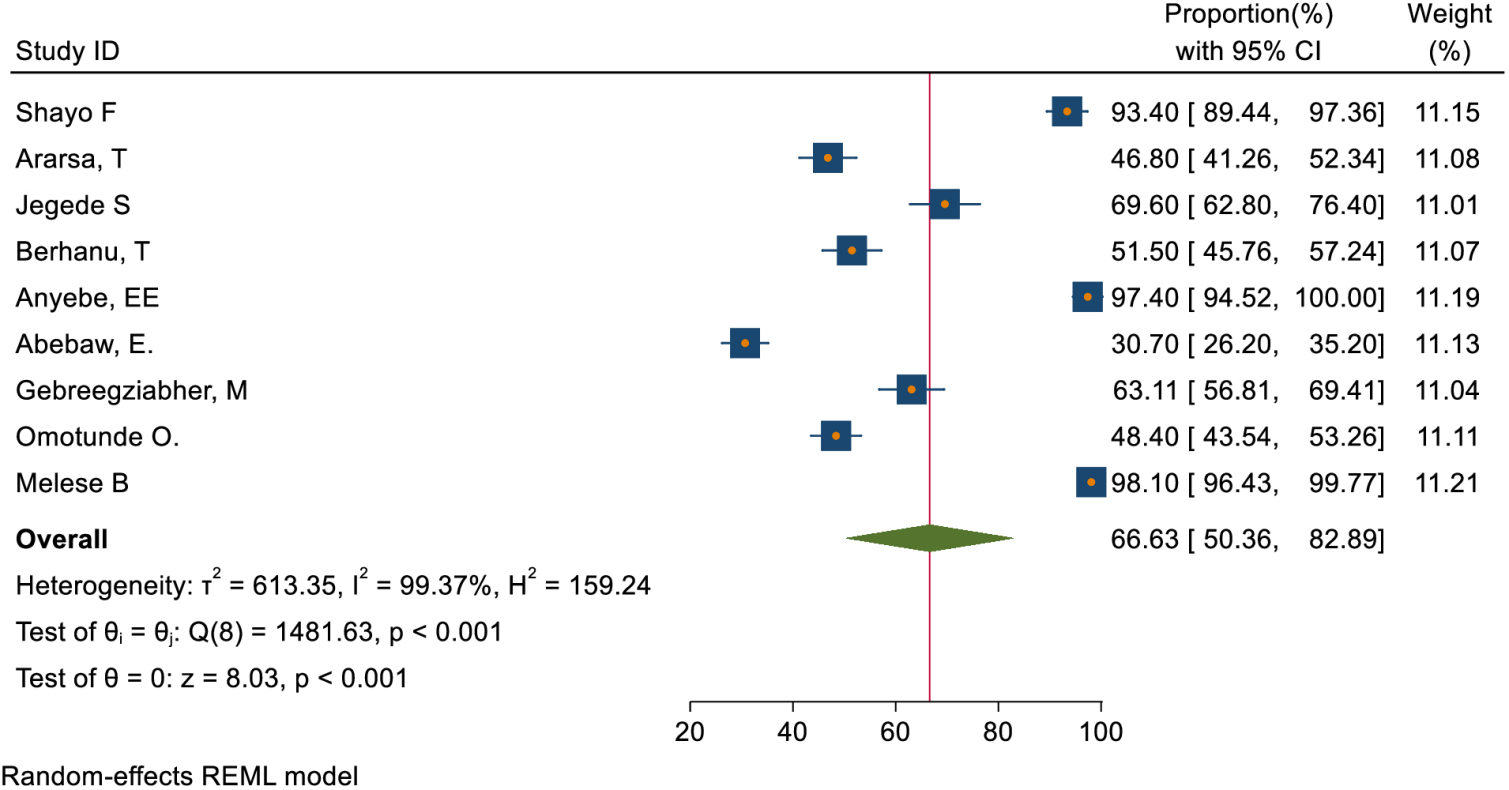
Overall magnitude of positive attitude of healthcare providers towards cervical cancer screening in Sub-Saharan Africa, 2023.

**Figure 9:**
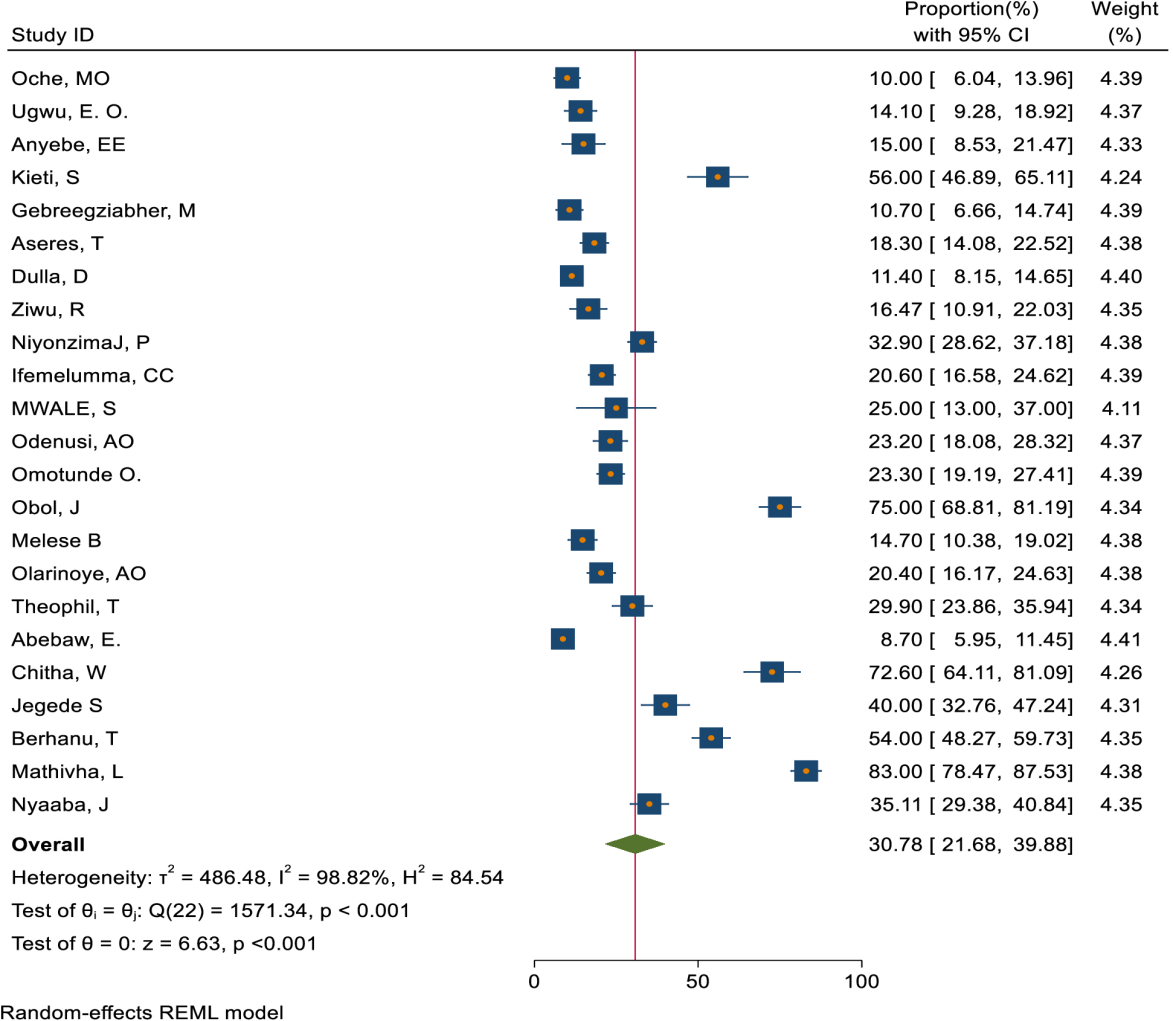
Overall magnitude of practice to ever screened for cervical cancer screening among female healthcare providers in Sub-Saharan Africa, 2023.

This study assessed knowledge status, attitude, and practice toward cervical cancer screening among healthcare providers in Sub-Saharan Africa. The pooled level of good knowledge towards cervical cancer screening was 49.68% (95% CI: 33.18–66.17) (**Figure 7**) whereas the pooled magnitude of positive attitude toward cervical cancer screening among healthcare providers in Sub-Saharan Africa was 66.63% (95% CI: 50.36– 82.89) (**Figure 8**). On the other hand, the pooled magnitude of ever screened for cervical cancer among female healthcare providers in Sub-Saharan Africa was 30.78% (95% CI: 21.69–39.88) (**Figure 9**). Since this result had a significant publication bias, the true pooled magnitude of female healthcare providers ever screened for cervical cancer was found to be 17.23% (95% CI; 6.08-28.37) after accounting for publication bias through trim and fill analysis.

## Subgroup analysis

**Figure 10:**
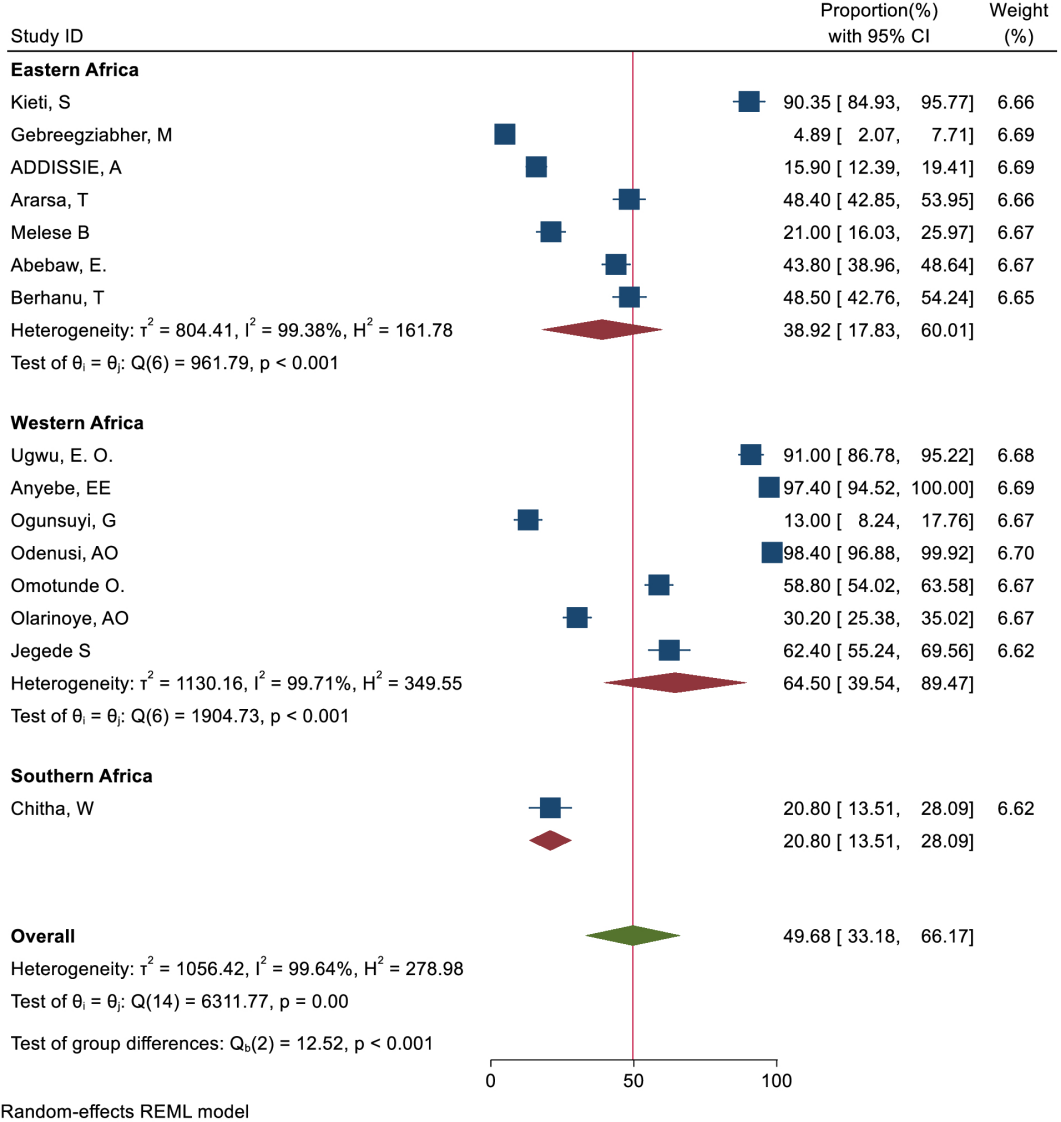
Subgroup analysis-based study area of knowledge status towards cervical cancer screening among health care providers in Sub-Sahara Africa, 2023.

**Figure 11:**
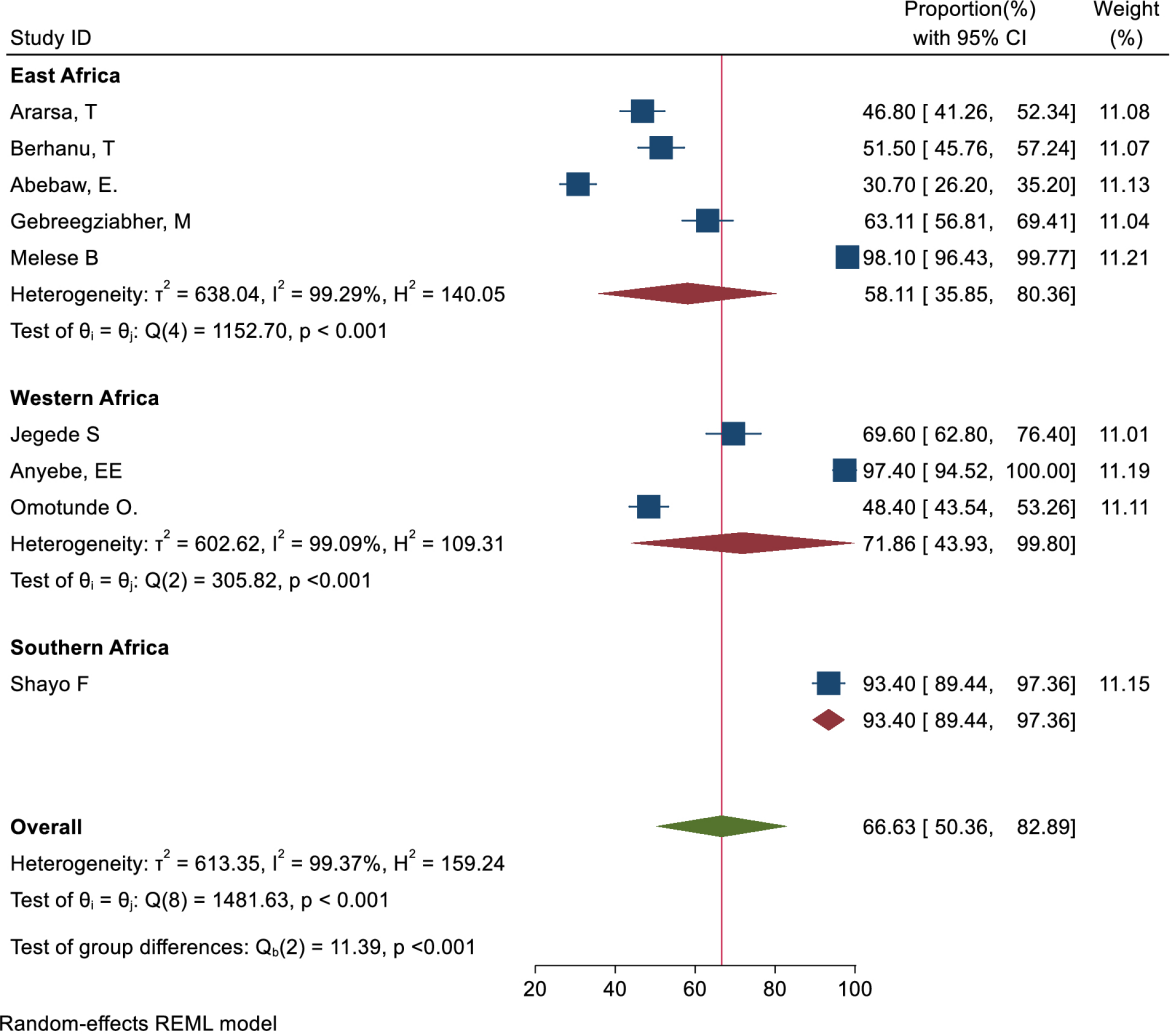
Sub group analysis-based study area of attitude status towards cervical cancer screening among healthcare providers in Sub Sahara Africa, 2023.

**Figure 12:**
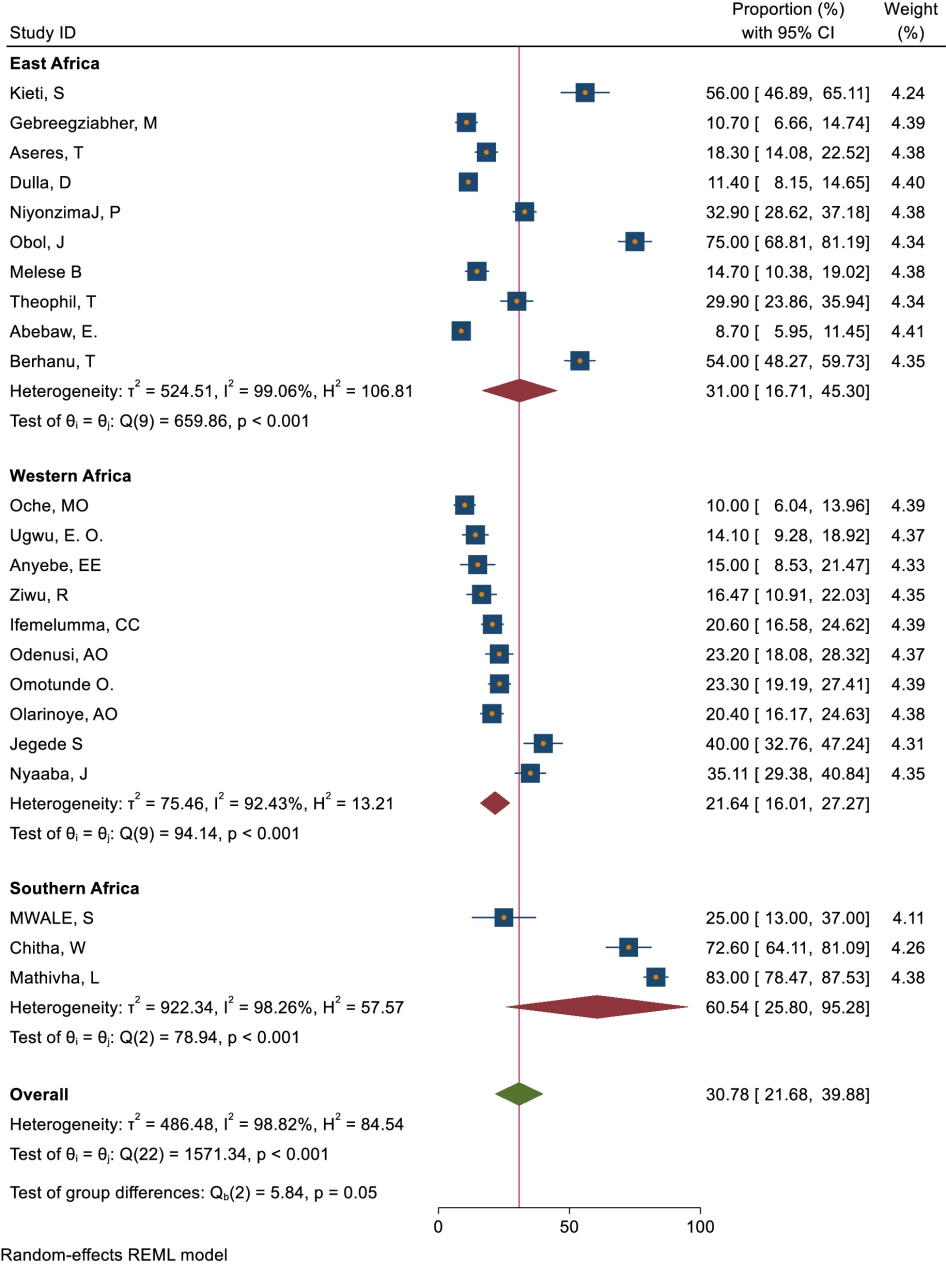
Sub group analysis-based on study area practices to ever screen for cervical cancer among healthcare providerd in Sub Sahara Africa, 2023.

Subgroup analysis was carried out based on geographical regions. Accordingly, the pooled magnitude of knowledge status towards cervical cancer screening among healthcare providers was 38.92% (95% CI: 17.83– 60.01) in East Africa, 64.50%(95% CI: 39.54–89.47) in Western Africa, and 20.80%(95% CI: 13.51– 28.09) in Southern Africa respectively (**Figure 10**). Similarly, the pooled magnitude of positive attitudes toward cervical cancer screening among healthcare providers was 58.11%(95% CI: 35.85– 80.36) in East Africa, 71.86%(95% CI: 43.93-99.80) in Western Africa, and 93.40%(95% CI: 89.44– 97.36) in Southern Africa respectively (**Figure 11**). Besides, the pooled magnitude of healthcare providers ever screened for cervical cancer was 31.00%(95% CI: 16.71– 45.30) in East Africa, 21.64%(95% CI: 16.01–27.27) in Western Africa, and 60.54%(95% CI: 25.80– 95.28) in Southern Africa respectively (**Figure 12**).

### Knowledge status of healthcare providers towards cervical cancer screening methods and regular cervical cancer screening interval

**Figure 13:**
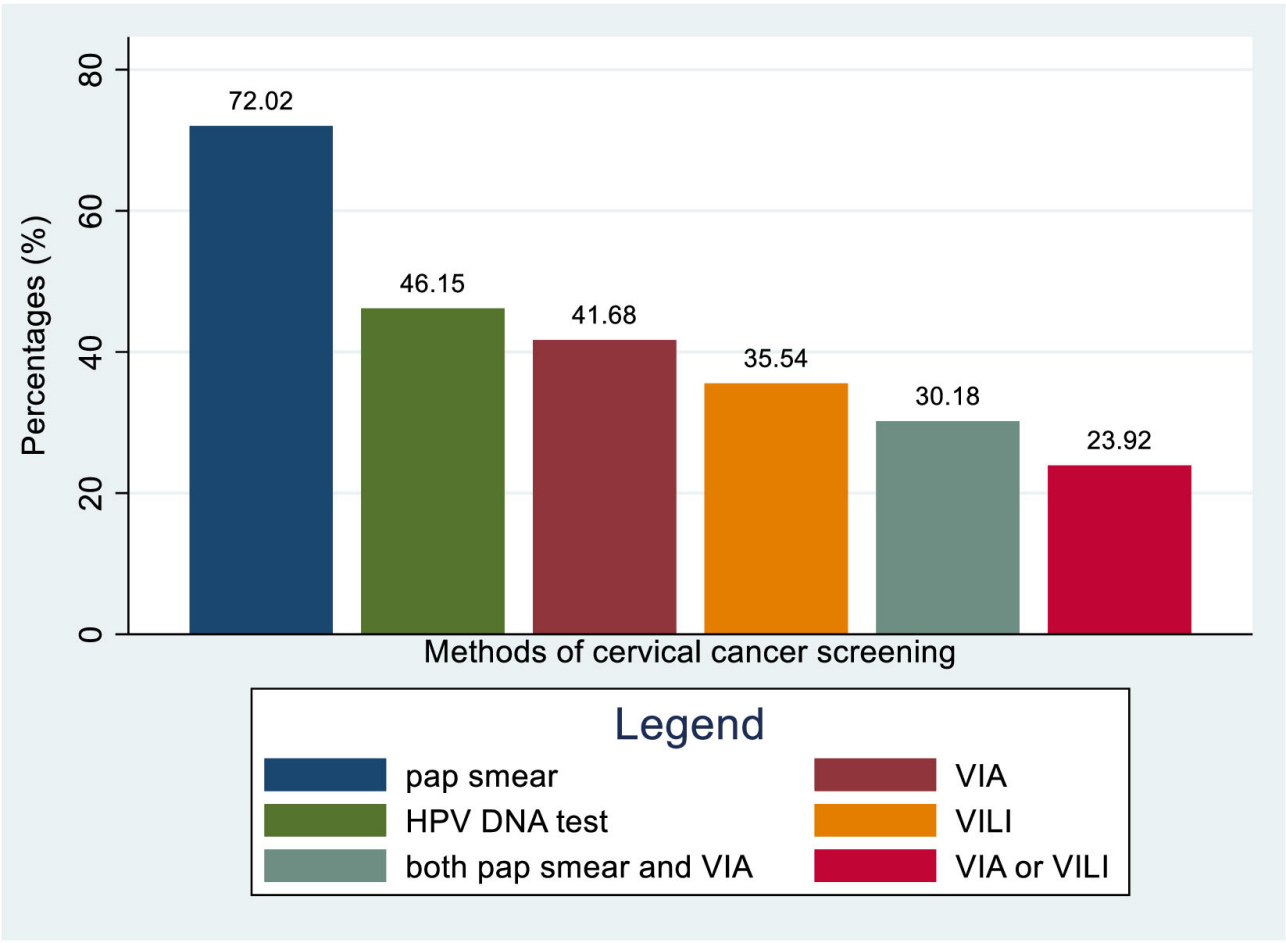
Bar graph for knowledge status of healthcare providers towards different cervical cancer screening methods in Sub-Sahara Africa, 2023.

**Figure 14:**
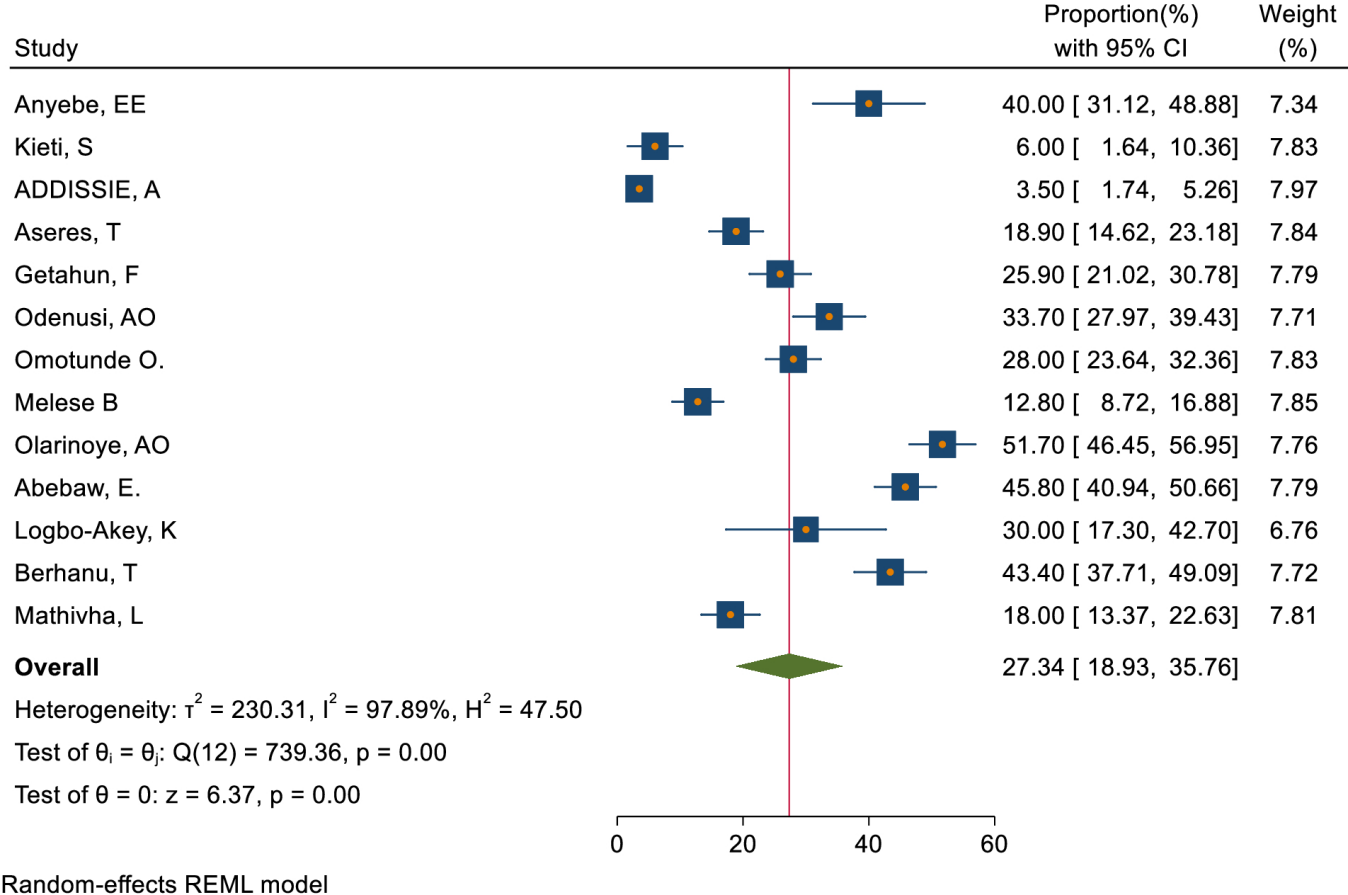
Overall knowledge status of healthcare providers towards knowing the regular interval for cervical cancer screening in Sub-Sahara Africa, 2023.

Around 72.02% of healthcare providers knew about pap smear as a screening method for cervical cancer, while 46.15% were aware of the HPV DNA test. Furthermore, approximately 41.68% knew about both pap smear and VIA, while 35.54% were only aware of VIA. A total of 30.18% were aware of VILI, and 23.92% knew about either VIA or VILI as screening methods for cervical cancer (**Figure 13**). The overall knowledge status towards knowing the regular interval for cervical cancer screening was 27.34% (95% CI: 18.93– 35.76) (**Figure 14**).

### Reasons for not being screened yet for cervical cancer

**Figure 15:**
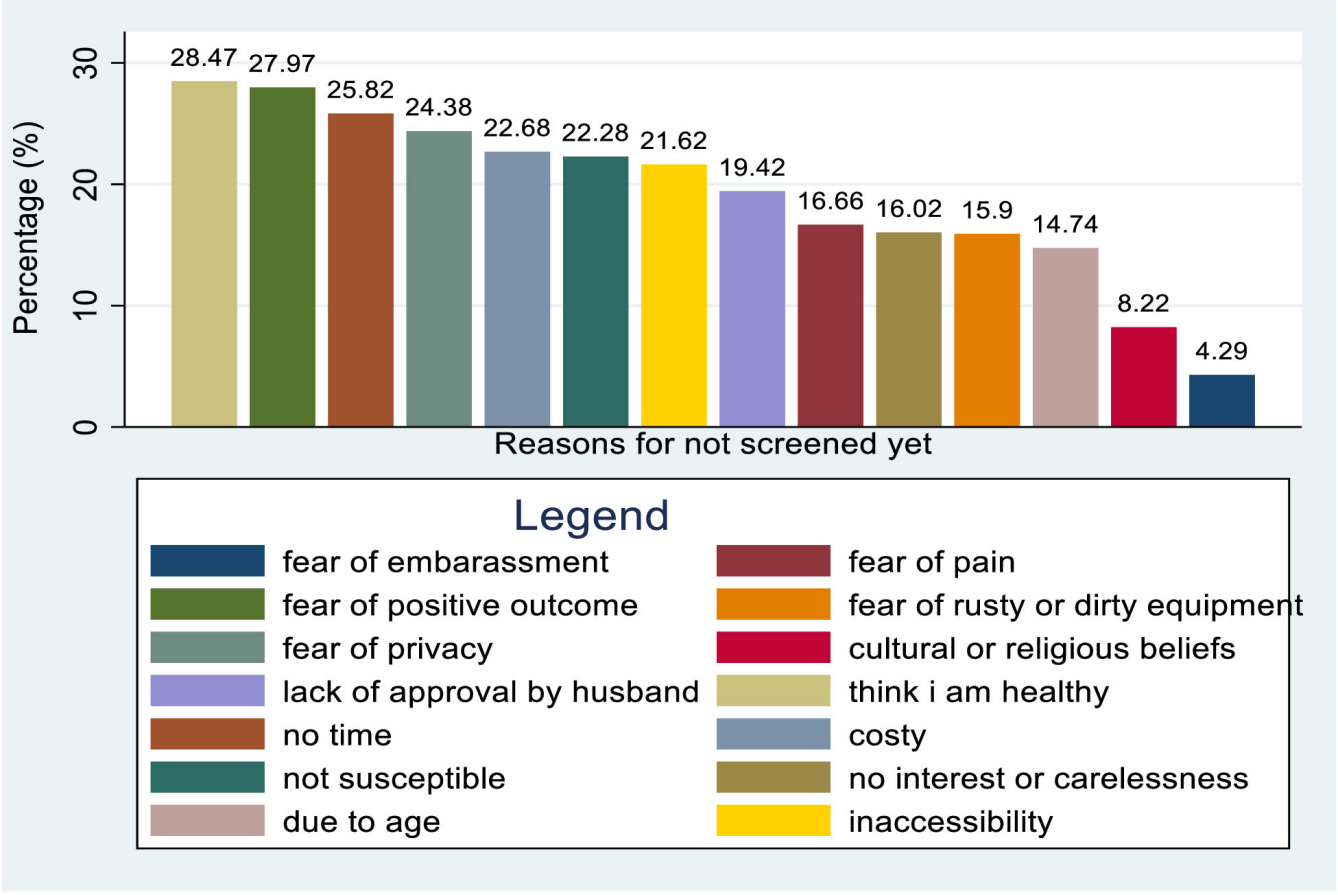
Bar graph indicating reasons for not screened yet for cervical cancer of health care providers towards risk factors of cervical cancer in Sub Sahara Africa, 2023.

A variety of reasons were given by respondents for not being screened. Approximately 28.47% of respondents chose not to get screened because they believed they were healthy. Another 27.97% were afraid of receiving a positive result, while 25.82% were afraid of experiencing pain during the screening. Additionally, 24.38% cited privacy concerns, 22.68% mentioned the cost of screening as a deterrent, 22.28% believed they were not susceptible to the condition being screened for, and 21.62% stated that the service was inaccessible. On the other hand, 19.42% of respondents were not screened due to their husband’s disapproval, 16.66% cited a lack of time, and 16.02% expressed no interest or carelessness. Other reasons included fear of using rusty or dirty equipment (15.9%), being considered too old for screening (14.74%), cultural or religious beliefs (8.22%), and fear of embarrassment (4.29%) (**Figure 15**).

### Publication bias assessment and sensitivity analysis

**Figure 16:**
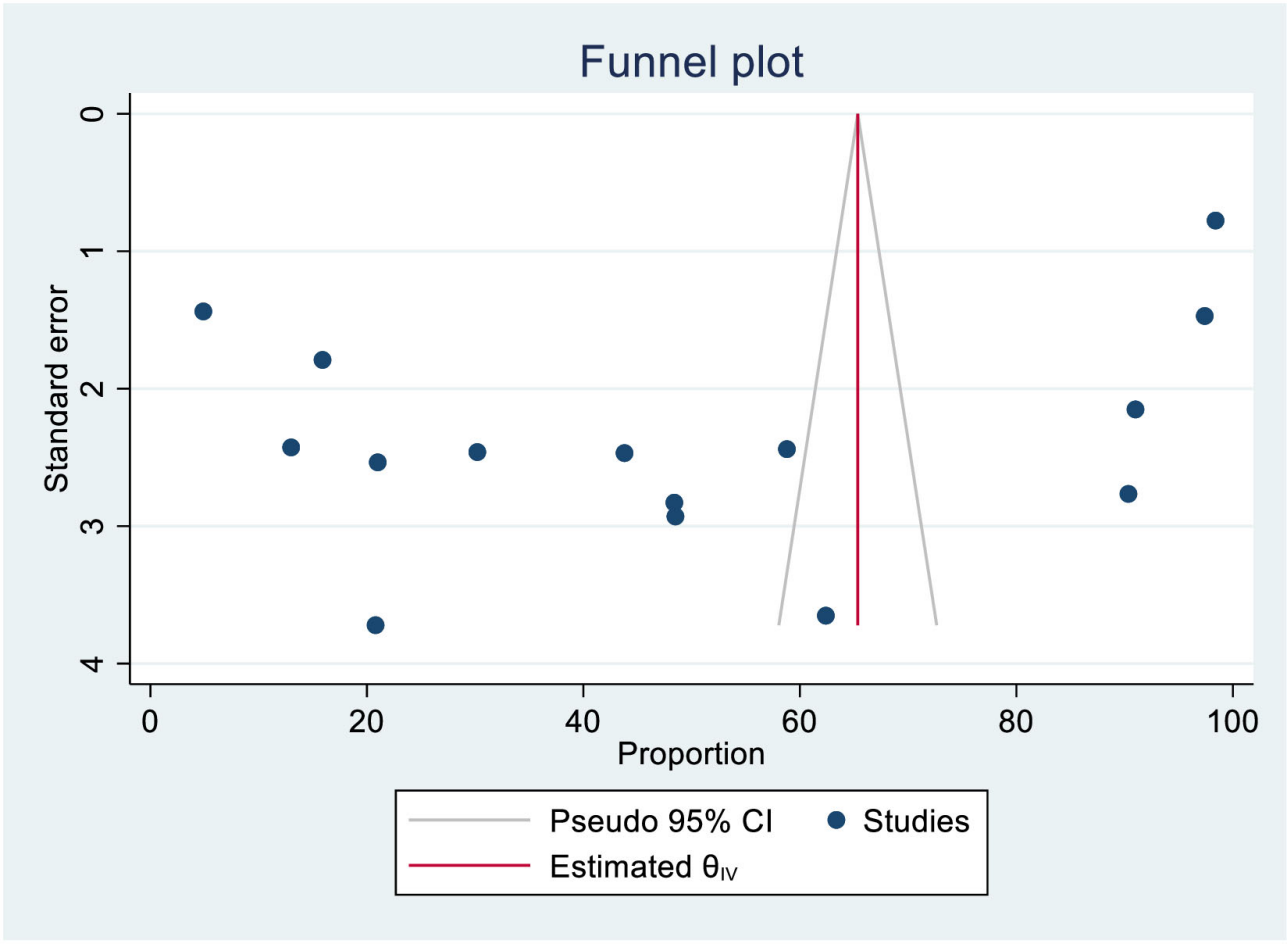
Funnel plot assessment for knowledge status towards cervical cancer screening among healthcare providers in Sub Saharan Africa, 2023.

**Figure 17:**
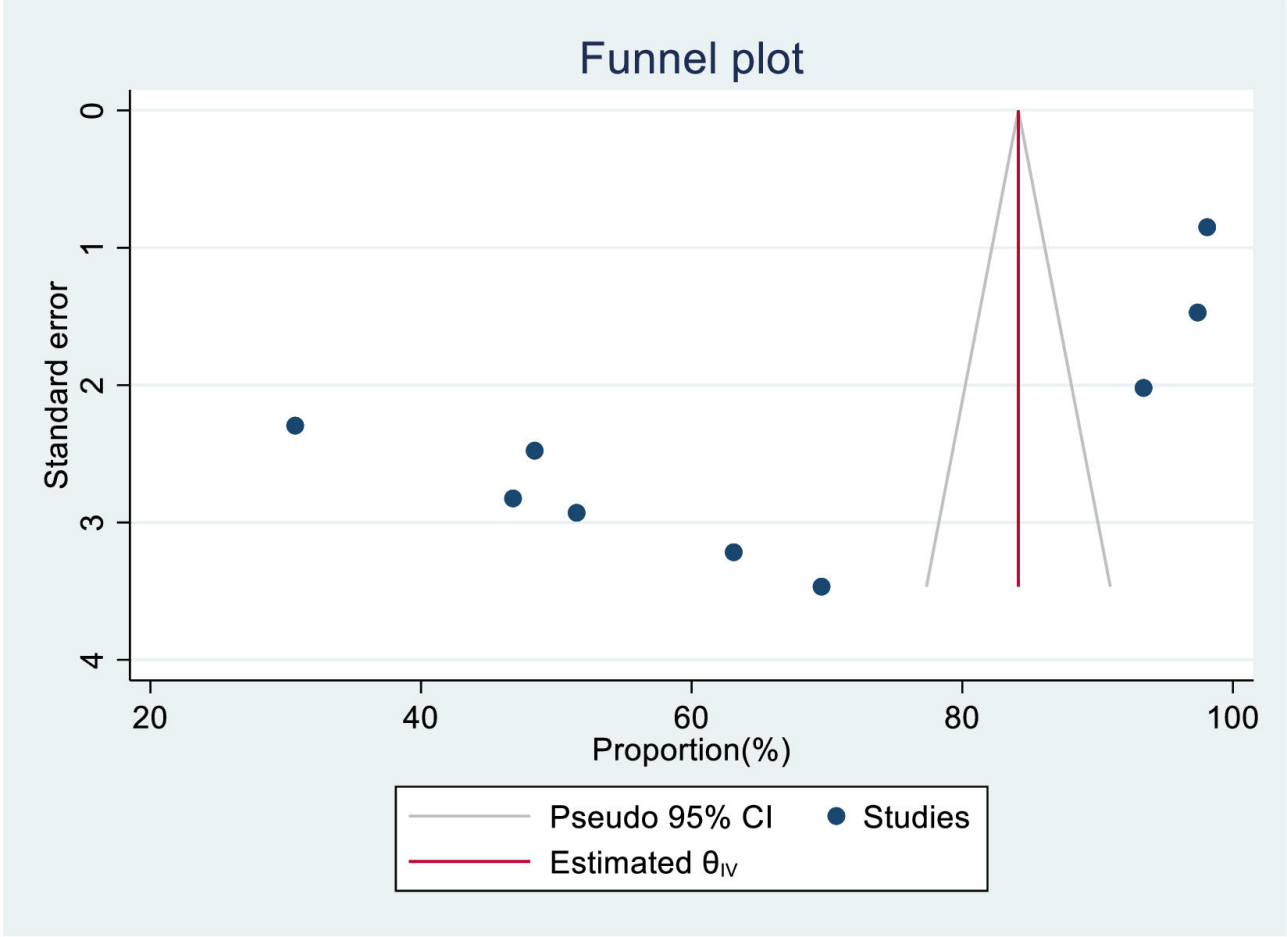
Funnel plot assessment for attitude status towards cervical cancer screening among health care providers in Sub Saharan Africa, 2023.

The researchers checked for publication bias by visually inspecting a funnel plot, as well as using statistical tests. The funnel plot showed that the included studies were distributed symmetrically. Moreover, both Begg’s and Egger’s tests indicated the absence of publication bias in the knowledge status of healthcare providers towards cervical cancer screening. The tests showed no statistical evidence of publication bias with a p-value greater than 0.05 (P value; Eggers test=0.38, Beggs test=1.00), and the funnel plot was symmetrical (**Figure 16**). On the contrary, the study found that there was publication bias in the data on healthcare providers’ attitudes towards cervical cancer screening. The funnel plot revealed an uneven distribution of the studies (**Figure 17**). The Egger’s test found evidence of publication bias (P value=0.04), indicating that there may be a bias in the published studies. However, Begg’s test (P value=0.60) suggested that there is no publication bias regarding the attitude of healthcare providers towards cervical cancer screening. To address this bias, the researchers conducted a trim and fill analysis however the pooled magnitude of attitude did not impute additional studies and the overall magnitude of attitude did not vary from the original findings.

**Figure 18:**
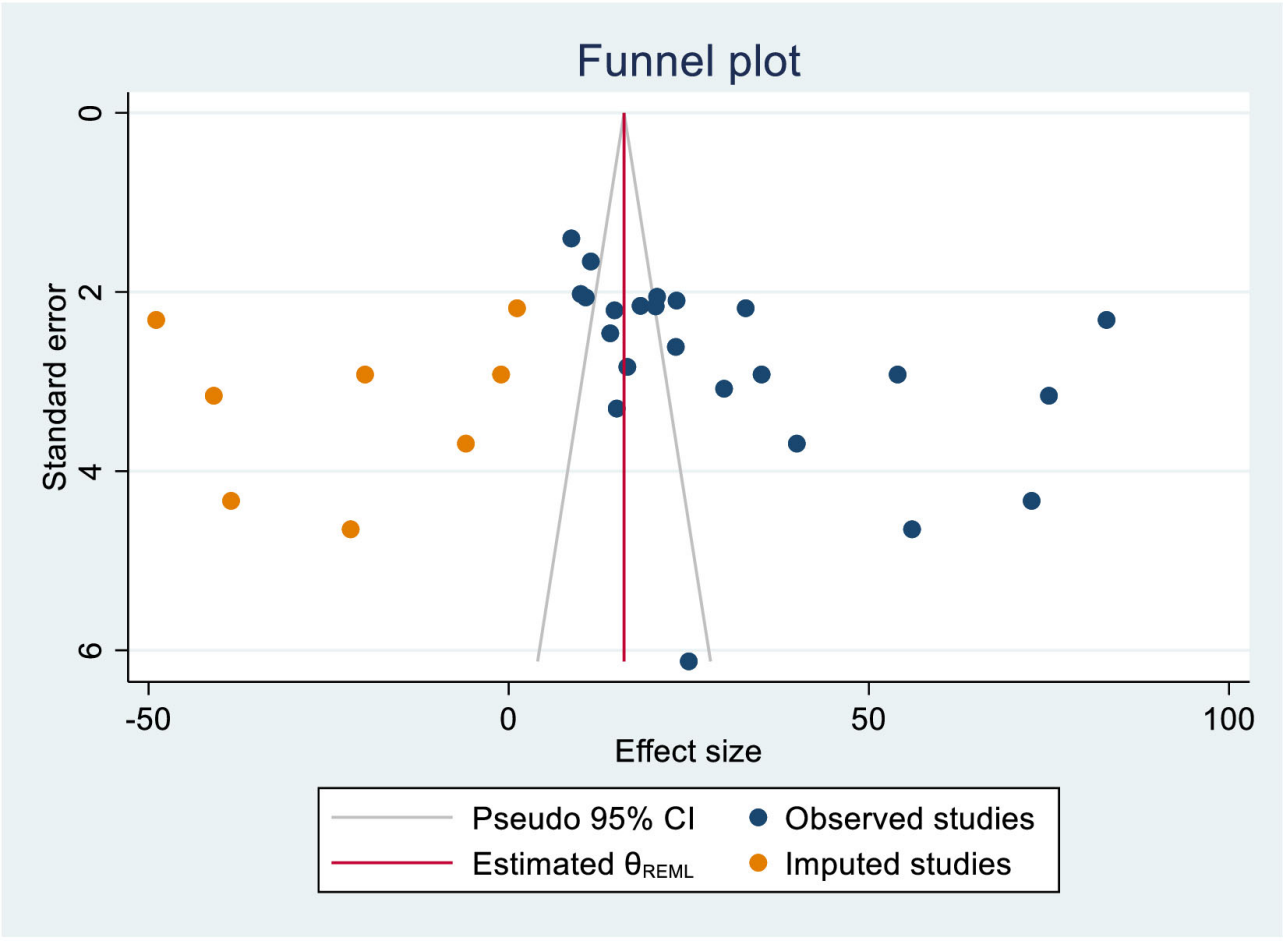
Funnel plot assessment of practice for female healthcare providers to ever screened status towards cervical cancer screening in Sub Saharan Africa, 2023.

**Figure 19:**
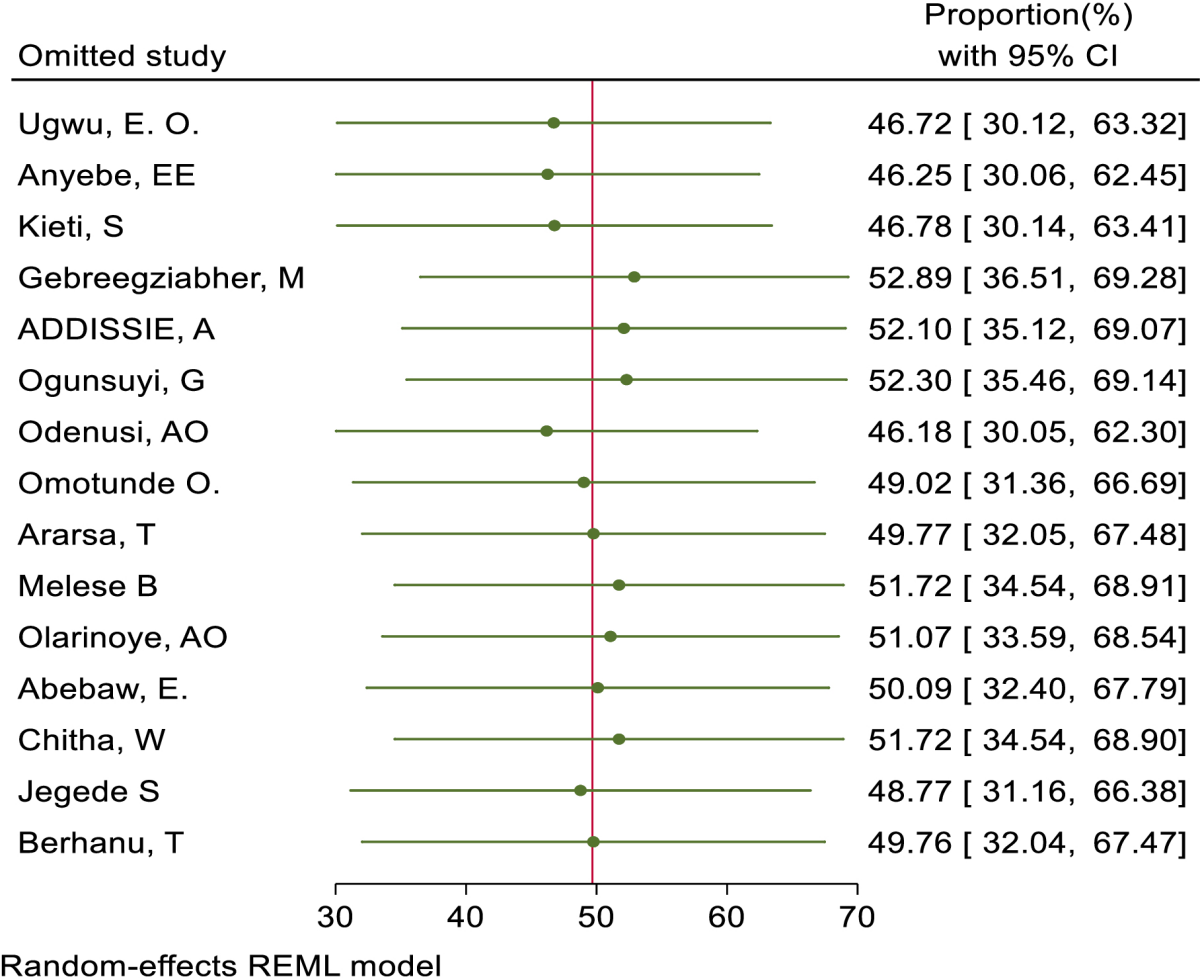
Sensitivity analysis for good knowledge status of health care providers towards cervical cancer screening among health care providers in Sub Saharan Africa, 2023.

**Figure 20:**
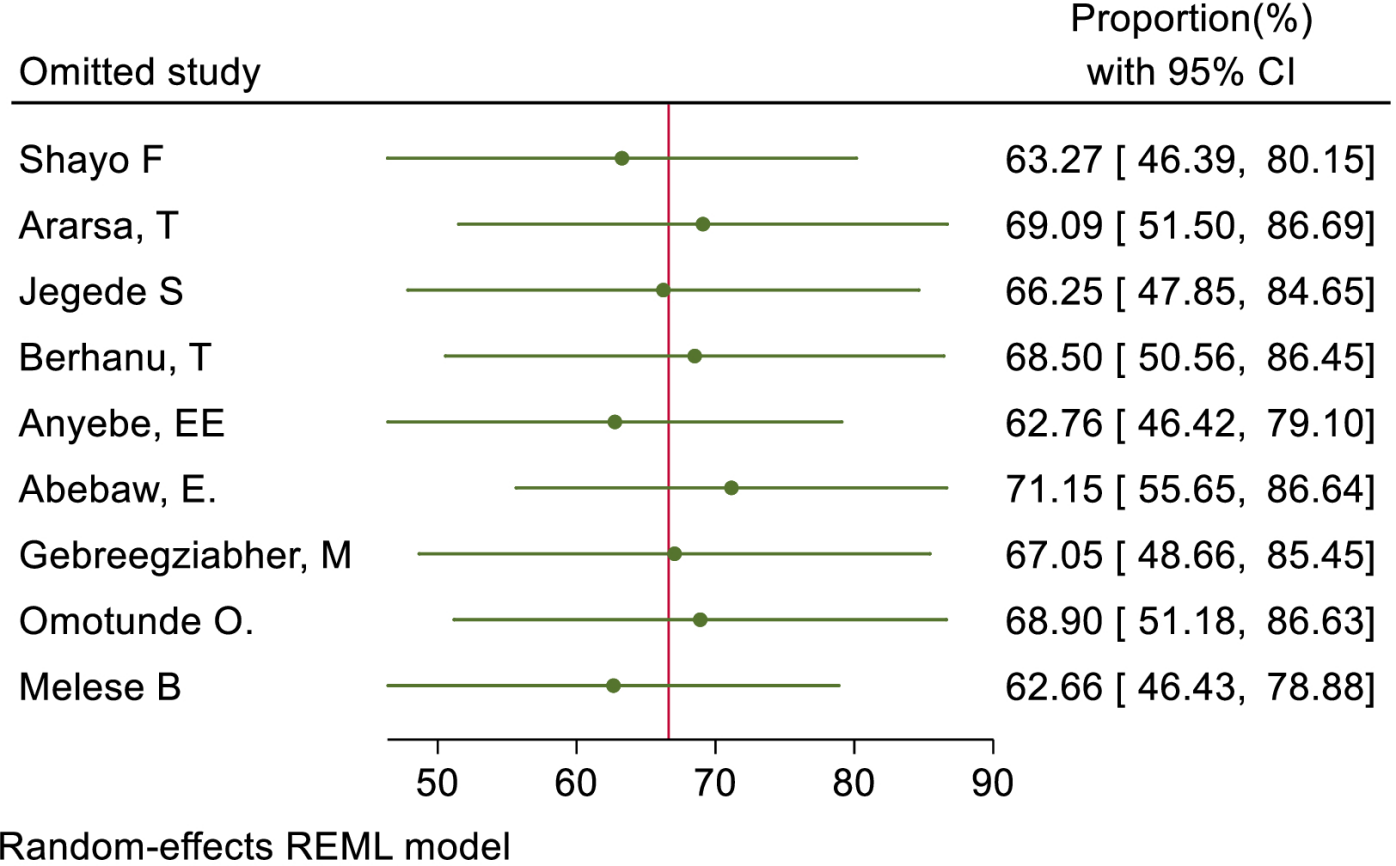
Sensitivity analysis for positive status of respondents towards cervical cancer screening among health care providers in Sub Saharan Africa, 2023.

**Figure 21:**
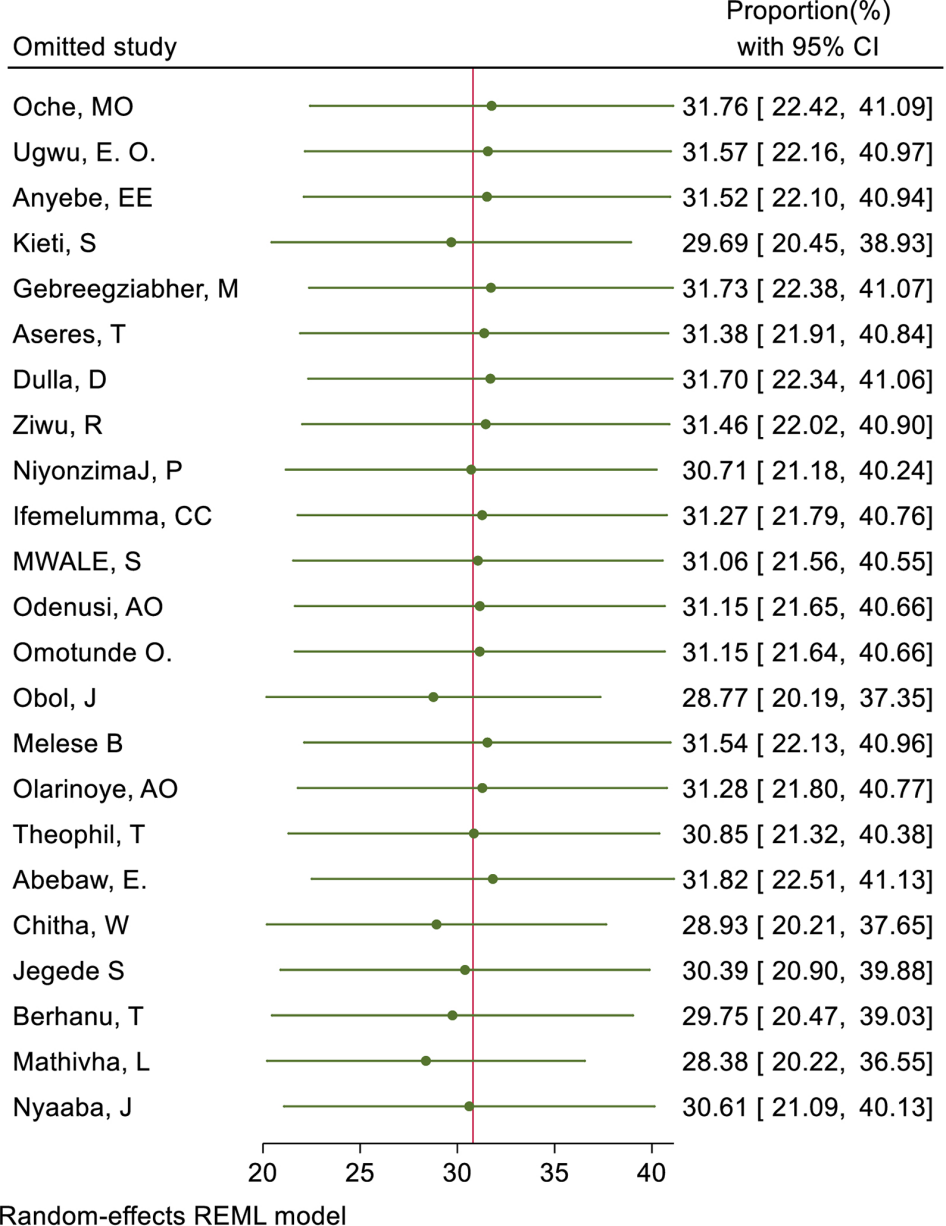
Sensitivity analysis for ever screened practice of female healthcare providers towards cervical cancer in Sub Sahara Africa, 2023.

We also assessed publication bias for the practice of female healthcare providers to ever screened for cervical cancer. Accordingly, Egger’s tests indicated the absence of publication bias in the pooled magnitude healthcare providers ever screened for cervical cancer (P value; Eggers test=0.10). However, Beggs tests showed statistical evidence of publication bias with a p-value less than 0.05(P value; Beggs test=0.04), and the funnel plot was asymmetrical (**Figure 18**). To address this bias, the researchers conducted a trim and fill analysis, which adjusted the original pooled results of 30.80%(95% CI: 21.69–39.91). The adjusted pooled magnitude of healthcare providers ever screened for cervical cancer was 17.23 (95% CI; 6.08-28.37) after including eight additional studies on the left side. This adjusted result is considered to be a more accurate representation of the true practice of healthcare providers ever screened for cervical cancer. The result of sensitivity analyses revealed that none of the studies included influenced the overall estimate (**Figures 19**–**21**).

### Meta-regression

Besides, trim and fill analysis for publication bias, meta-regression analysis was performed by considering sample size and publication year for the included studies to identify sources of bias for the pooled prevalence. We included a total of 9 studies (34, 35, 47–50, 64, 66, 67) for attitude towards cervical cancer screening and 23 studies (23, 34, 35, 45–49, 53–60, 62, 64–66, 68–70) for the practice of ever being screened for cervical cancer in our meta-regression analysis. In this analysis, the sample size was a statistically significant source of publication bias for the pooled magnitude of attitude status whereas publication year is the source of publication bias for health care providers’ practice to ever screen for cervical cancer as shown in the values from the meta-regression analysis (**Table 3**).

**Table 3:** Meta-regression of attitude and practice of cervical cancer screening among health care providers in Sub Saharan Africa, 2023.

Domain	Variables	Coefficient	LCL	UCL	P-value
Attitude status	Publication year	1.80	-4.83	8.44	0.59
	Sample size used	-0.23	-0.39	-0.06	<0.01
Practice	Publication year	3.84	1.14	6.55	<0.01
	Sample size used	-0.05	-0.13	0.02	0.15

## Discussion

The World Health Assembly aimed to reduce cervical cancer through a 90-70-90 policy, but healthcare providers in sub-Saharan Africa had significant gaps in their knowledge, attitude, and practice towards cervical cancer and its screening. The results of this meta-analysis showed that the pooled magnitude of good knowledge status and positive attitude of healthcare providers towards cervical cancer was 67.93% (95% CI: 53.36–82.50) and 55.26% (95% CI: 34.28–76.23)respectively. This implied that healthcare providers do not have adequate knowledge and a positive attitude towards cervical cancer. This pooled magnitude of knowledge was consistent with 80% in Saudi Arabia (72) and 75.15% in India (73) and it was lower as compared to 92% in Pakistan (74). On the other hand, the pooled magnitude of positive attitudes towards cervical cancer screening was lower as compared to 78% in Pakistan (74). The possible explanation for these discrepancy in the magnitude of knowledge and attitudes towards cervical cancer could results from variations in training, workload, resource limitations, and cultural beliefs within the specific environments being studied. These factors may contribute to the observed differences in understanding and behaviors among individuals.

This review also found that the overall level of good knowledge, positive attitude, and practice of cervical cancer screening among healthcare providers was 49.68% (95% CI: 33.18–66.17), 66.3% (95% CI: 50.36–82.89), and 30.785% (95% CI: 21.68–39.88), respectively. The statement suggests that healthcare providers in sub-Saharan Africa have limited knowledge, attitude, and practice towards cervical cancer screening despite the fact that they are expected to serve as examples for eligible women who should undergo such screening. The overall magnitude of knowledge status towards cervical cancer screening was consistent with overall knowledge of 60.4% in Egypt (75) and 56.3% in China (76). However, it was lower as compared to 86.2% in India (73). Moreover, the overall magnitude of positive attitude status towards cervical cancer screening was also consistent with 53.4% in Egypt (75) but it was lower as compared to 85.47% in India (73) and 90% in Turkey (30). This discrepancy in healthcare providers’ knowledge and attitude towards cervical cancer screening could be attributed to differences in their specialization, experiences, and professional competency levels of health professionals included in their sample. These differences may also be influenced by variations in training and education provided to healthcare professionals in different countries. The limited access to screening services and lack of emphasis on cervical cancer screening in the study location may contribute to this discrepancy, which could be further influenced by variations in access to screening services and information dissemination.

The findings of this study regarding the overall practice of female healthcare providers who had been screened for cervical cancer in Sub-Saharan Africa was consistent with 12.7% in India (73), 26.2% in Saudi Arabia (77), and 26.6% in Sri Lankan (78). The result was not significantly different from a systematic review and meta-analysis conducted on women of reproductive age in Sub-Saharan Africa, where the percentage was 12.87% (79). But it was lower as compared with 42.2% in Qatar (80) and 45.2% in south Turkey (81). This variation might be justified by the extent of the studies and differences in the health service, sociodemographic characteristics of study participants, and setting and study period.

Female healthcare providers reported various reasons for not being screened for cervical cancer, including fear of embarrassment, fear of pain, lack of time, fear of positive outcomes, fear of privacy, lack of availability of services, carelessness, fear of using dirty or rusty equipment, lack of approval from their husbands, cultural and religious beliefs, thinking they are not susceptible to the disease, high costs, feeling healthy, and lack of access to services. This suggests that individuals who perceive themselves as healthy often overlook preventive services, and a lack of awareness about cervical cancer screening contributes to low utilization of these services. This finding was supported by a previous study conducted in India (82) and in Korea (83).

This review also found that 72.02% of healthcare providers know about pap smears as a way to screen for cervical cancer. Around 46.15% are aware of the HPV DNA test as a screening method, while 41.68% know about VIA and 35.54% know about VILI. Additionally, 30.18% are aware of both pap smears and VIA, and 23.92% are aware of either VIA or VILI as screening methods for cervical cancer. Healthcare providers in Sub-Saharan Africa are more knowledgeable and experienced in using pap smears for cervical cancer screening compared to other methods like VIA, VILI, or HPV tests. This is because pap smears have been widely used in healthcare facilities in the region. About 27.34% of healthcare providers were aware of the regular interval for cervical cancer screening. this finding is low as comparable with 39% in Pakistan (74). This difference could be explained by the variations in the sociodemographic characteristics of the study participants and the disparities quality of training they received.

A systematic review in Sub-Saharan Africa found that the COVID-19 pandemic has led to disruptions in cervical cancer screening, diagnosis, and treatment services due to factors such as transportation limitations, staff shortages, and patients’ fears of contracting the virus. To address these challenges, telemedicine and virtual platforms have been utilized for patient consultations and follow-ups during the pandemic in the region (84).

## Strengths and limitations of the study

The strengths of this review include being the first to examine the knowledge, attitude, and practice of health providers in sub-Saharan Africa regarding cervical cancer screening. Additionally, the review followed PRISMA guidelines and incorporated both published and unpublished research. However, a limitation of the review is that only studies written in English were included in the review.

## Conclusions and recommendation

The level of knowledge, attitude, and practice of cervical cancer screening among healthcare providers in Sub-Saharan Africa was suboptimal. Healthcare providers need to take an active role in promoting women’s health and preventing disease. This involves ensuring that healthcare professionals are knowledgeable about cervical cancer and its screenings, as well as having a positive outlook towards screening and being screened themselves. It is important to educate healthcare providers about misconceptions regarding cervical cancer screening and to increase awareness of the availability of these services in various centers across the country. Given the impact that their knowledge, attitude and practice can have on a large number of clients, it is imperative that swift action be taken. This could include providing training to participants to enhance their understanding, influence their beliefs, and encourage more individuals to utilize screening services. These efforts will help reduce the occurrence of cervical cancer among high-risk women. Hence, Policymakers as well as program implementers need to enhance the level of knowledge, attitude status, and screening habits of healthcare providers towards cervical cancer. Thus, healthcare providers should be role models for all other women for those services delivered to patients.

## Data Availability

The original contributions presented in the study are included in the article/supplementary material. Further inquiries can be directed to the corresponding author.
